# Mule deer fawn recruitment dynamics in an energy disturbed landscape

**DOI:** 10.1002/ece3.9976

**Published:** 2023-04-19

**Authors:** Kilian J. Murphy, David R. Roberts, William F. Jensen, Scott E. Nielsen, Sandra K. Johnson, Brian M. Hosek, Bruce Stillings, Jesse Kolar, Mark S. Boyce, Simone Ciuti

**Affiliations:** ^1^ Laboratory of Wildlife Ecology and Behaviour, School of Biology and Environmental Science University College Dublin Dublin Ireland; ^2^ Ministry of Environment and Parks, Government of Alberta 3535 Research Road NW Calgary Alberta T2L 2K8 Canada; ^3^ InnoTech Alberta 3608 33 Street NW Calgary Alberta T2 L 2A6 Canada; ^4^ North Dakota Game and Fish Department Bismarck North Dakota 58501 USA; ^5^ Department of Renewable Resources University of Alberta Edmonton Alberta Canada; ^6^ North Dakota Game and Fish Department Dickinson North Dakota 58601 USA; ^7^ Department of Biological Sciences University of Alberta Edmonton Alberta Canada

**Keywords:** disturbance, predator‐prey, game management, mule deer, wildlife management, energy development, recruitment, mule deer fawn survival, harsh weather

## Abstract

Wildlife population dynamics are modulated by abiotic and biotic factors, typically climate, resource availability, density‐dependent effects, and predator–prey interactions. Understanding whether and how human‐caused disturbances shape these ecological processes is helpful for the conservation and management of wildlife and their habitats within increasingly human‐dominated landscapes. However, many jurisdictions lack either long‐term longitudinal data on wildlife populations or measures of the interplay between human‐mediated disturbance, climate, and predator density. Here, we use a 50‐year time series (1962–2012) on mule deer (*Odocoileus hemionus*) demographics, seasonal weather, predator density, and oil and gas development patterns from the North Dakota Badlands, USA, to investigate long‐term effects of landscape‐level disturbance on mule deer fawn fall recruitment, which has declined precipitously over the last number of decades. Mule deer fawn fall recruitment in this study represents the number of fawns per female (fawn:female ratio) that survive through the summer to October. We used this fawn recruitment index to evaluate the composite effects of interannual extreme weather conditions, energy development, and predator density. We found that density‐dependent effects and harsh seasonal weather were the main drivers of fawn fall recruitment in the North Dakota Badlands. These effects were further shaped by the interaction between harsh seasonal weather and predator density (i.e., lower fawn fall recruitment when harsh weather was combined with higher predator density). Additionally, we found that fawn fall recruitment was modulated by interactions between seasonal weather and energy development (i.e., lower fawn fall recruitment when harsh weather was combined with higher density of active oil and gas wells). Interestingly, we found that the combined effect of predator density and energy development was not interactive but rather additive. Our analysis demonstrates how energy development may modulate fluctuations in mule deer fawn fall recruitment concurrent with biotic (density‐dependency, habitat, predation, woody vegetation encroachment) and abiotic (harsh seasonal weather) drivers. Density‐dependent patterns emerge, presumably due to limited quality habitat, being the primary factor influencing fall fawn recruitment in mule deer. Secondarily, stochastic weather events periodically cause dramatic declines in recruitment. And finally, the additive effects of human disturbance and predation can induce fluctuations in fawn fall recruitment. Here we make the case for using long‐term datasets for setting long‐term wildlife management goals that decision makers and the public can understand and support.

## Graphical abstract

We use a 50 years time series to investigate the relative roles of population ecology, landscape change, climate, predation, and human disturbance on mule deer population recruitment in a changing landscape. We found that density‐dependent effects and quality habitat availability were the main drivers of mule deer recruitment but interactions with severe seasonal weather, predation, and oil and gas development modulated recruitment in a more subtle way. Approximately, we demonstrate the value of long‐term data collection and formal data analysis to disentangle the drivers of wildlife communities in a changing world for evidence‐based policy and management of landscapes and wildlife.

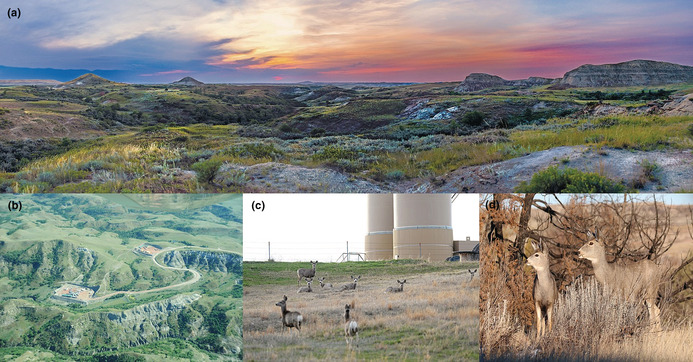

## 1 INTRODUCTION

Human disturbances to wildlife can cause biodiversity loss, habitat fragmentation and changes to animal behavior (Carrasco et al., 2009; Hallmann et al., 2017; Loehle & Eschenbach, 2011). Understanding the mechanism of wildlife response to disturbance can aid the conservation of species subjected to human pressures. Human modification of ecosystems and fragmentation of habitats yields trophic responses across ecological communities as it forces top‐down and bottom‐up cascading effects on predators and prey (Karakoç et al., 2018). While ecosystems are inherently dynamic, rapid anthropogenic change has forced wildlife to occupy habitats alien to their evolutionary history resulting in changes to population dynamics across species (Guiden et al., 2019). Unfortunately, there is a lack of understanding of multiple effects of human disturbance on wildlife and their ecology. This is in some part due to a lack of long‐term, systematic monitoring schemes needed to detect these trends and longitudinal studies required to identify the cause of ecosystem change in response to anthropogenic activity (Graham et al., 2005; Murphy et al., 2022).

Expanding energy development is a primary source of human disturbance to wildlife worldwide (Allred et al., 2015; Copping et al., 2014; Northrup & Wittemyer, 2013; Trainor et al., 2016), altering habitat use and recruitment in rangeland species (Gamo & Beck, 2017; Sawyer et al., 2002; Sawyer & Lindzey, 2004; Sawyer et al., 2006; Sawyer et al., 2009). Energy development alters habitat composition by changing soil and vegetation structure, increasing habitat fragmentation, and altering natural vegetation (Naugle et al., 2011; Northrup & Wittemyer, 2013; Souther et al., 2014; Walker et al., 2019). The effect of disturbance associated with energy development is most significant for habitat specialists and area‐sensitive species that require large expanses of intact habitat (e.g., Brittingham et al., 2014; Walker et al., 2019). Up to 75% of habitat loss associated with energy development can be attributed to supporting infrastructure such as roads, fences, and pipelines (Baynard et al., 2017; Copeland et al., 2017; Donnelly et al., 2017; Preston & Kim, 2016; Sawyer et al., 2002) resulting in biodiversity decline for multiple taxa in proximity to these features (Benítez‐López et al., 2010).

Human disturbance to wildlife can introduce novelty into ecosystem communities and alter predator–prey interactions by influencing resource availability and modifying habitat that modulates spatial overlap, such as reducing refugia for prey species (Guiden et al., 2019; Muhly et al., 2011). How a disturbance influences predator and prey communities is dependent on the nature of the behavioral response of each group. For example, prey can benefit when predators are displaced from modified landscapes (Burr, 2014). Conversely, predators can benefit from landscape modifications that increase hunt success (Berger, 2007; Dickie et al., 2020). If both predator and prey change behaviors to avoid human activity, both species can be pushed into ever‐shrinking natural habitats, where prey must alter their behavior to avoid predators, e.g., increase vigilance and other predation risk avoidance behaviors (Ciuti et al., 2012; Murphy et al., 2021). These trade‐offs incur a fitness cost that can be additive to other environmental constraints. For instance, harsh seasonal weather and reduced resource availability can cause additional negative impacts to survival and recruitment of prey populations (Basille et al., 2013; Murphy et al., 2021). Therefore, it is important to disentangle the drivers of prey population dynamics within modified ecosystems, specifically the interplay between human disturbance, predation, and seasonal environmental change.

Without long‐term studies on the effect of disturbance to wildlife, relatively short‐term consequences of long‐term effects can be misidentified that may result in human–wildlife conflict or further disturbance (Ballard et al., 2001; Graham et al., 2005; Murphy et al., 2022; Woodroffe et al., 2005). Thus, up‐to‐date knowledge on population productivity is important to monitor long‐term population health. Recruitment can be a proxy of prey population productivity because it represents the joint contribution of fecundity and offspring survival, making it one of the most variable parameters in large herbivore populations and a key factor affecting population growth rates (Gaillard et al., 1998). Many metrics exist to examine population productivity in ungulates. For instance, adult survival is often stable whereas fecundity and recruitment have greater annual variability and have been shown to be better predictors of long‐term population productivity (Gaillard et al., 1998). Recruitment is most commonly affected by climate, habitat quality, competition, and predation and may be particularly sensitive to human disturbance (Vye et al., 2017; Whittaker & Lindzey, 1999). Recruitment is also of particular interest for the conservation of hunted ungulate populations, for instance, deer species, because it can dictate harvest quotas (Ciuti et al., 2015). Consequently, studies on harvested deer populations are essential for monitoring trends and that might arise from limiting factors such as predation or disturbance (Ballard et al., 2001).

By using one of the longest term surveys of wild cervids in North America (1962–2012) accompanied by unique high‐resolution spatiotemporal data on oil and gas development, we aim to disentangle the biotic (e.g., population density and predator density) and abiotic (e.g., seasonal weather and habitat quality) drivers of mule deer (*Odocoileus hemionus*) fawn fall recruitment in the North Dakota Badlands. Because of the unique data on oil and gas developments, we had the opportunity to unravel whether predation by coyotes and harsh seasonal weather influenced fawn fall recruitment when interacting with long‐term disturbance including a period of rapidly escalating landscape modification, i.e., rapid development of hydraulic fracturing for oil and gas (NDDMR, 2021). Mule deer fawn fall recruitment in this study represents the number of fawns per female (fawn:female.ratio) that survive to October. We used this as a proxy for fawns surviving the first 4–6 months of life where risks such as abortion, malnutrition, drought, and predation are prevalent. While fawn:female ratio in fall is a strong index of interannual fawn summer survival, it is important to state that only a portion of this population will survive the overwinter period and contribute to the adult population (Forrester & Wittmer, 2013). Our a priori hypothesis was that long‐term energy disturbance would negatively affect fall fawn recruitment when combined with harsh seasonal weather and/or predation pressure, although we did not have a priori expectations on the effect size of such interactions. Our goal was to describe the long‐term effects of energy development on herbivore ecology within a landscape with increasing human‐developed disturbances.

## 2 METHODS

We used generalized linear mixed‐effect additive models (Wood, 2017) to explain the variability of mule deer fawn recruitment (fall fawn:female ratios) as a function of environmental (weather, landscape), ecological (predator, habitat), and anthropogenic disturbance (energy development, associated infrastructure) variables across 26 study sites and 50 years of monitoring in the North Dakota Badlands. We used the *psych* package (Revelle, 2021) in R to calculate Pearson's correlation coefficients for all predictor combinations, and we wished to use in the model and excluded collinear smooth terms (Dormann et al., 2013; Wood, 2017). All analyses were conducted in R 4.0.2 (R Development Core Team, 2021). Our observational procedures were part of a study plan approved by the North Dakota Game and Fish Department and U.S. Fish and Wildlife Service Federal Aid in Wildlife Restoration that did not involve animal handling and adhered to relevant regulations and guidelines regarding the ethics of animal welfare (Sikes, & Animal Care and Use Committee of the American Society of Mammalogists, 2016).

### 2.1 Study area

**FIGURE 1:**
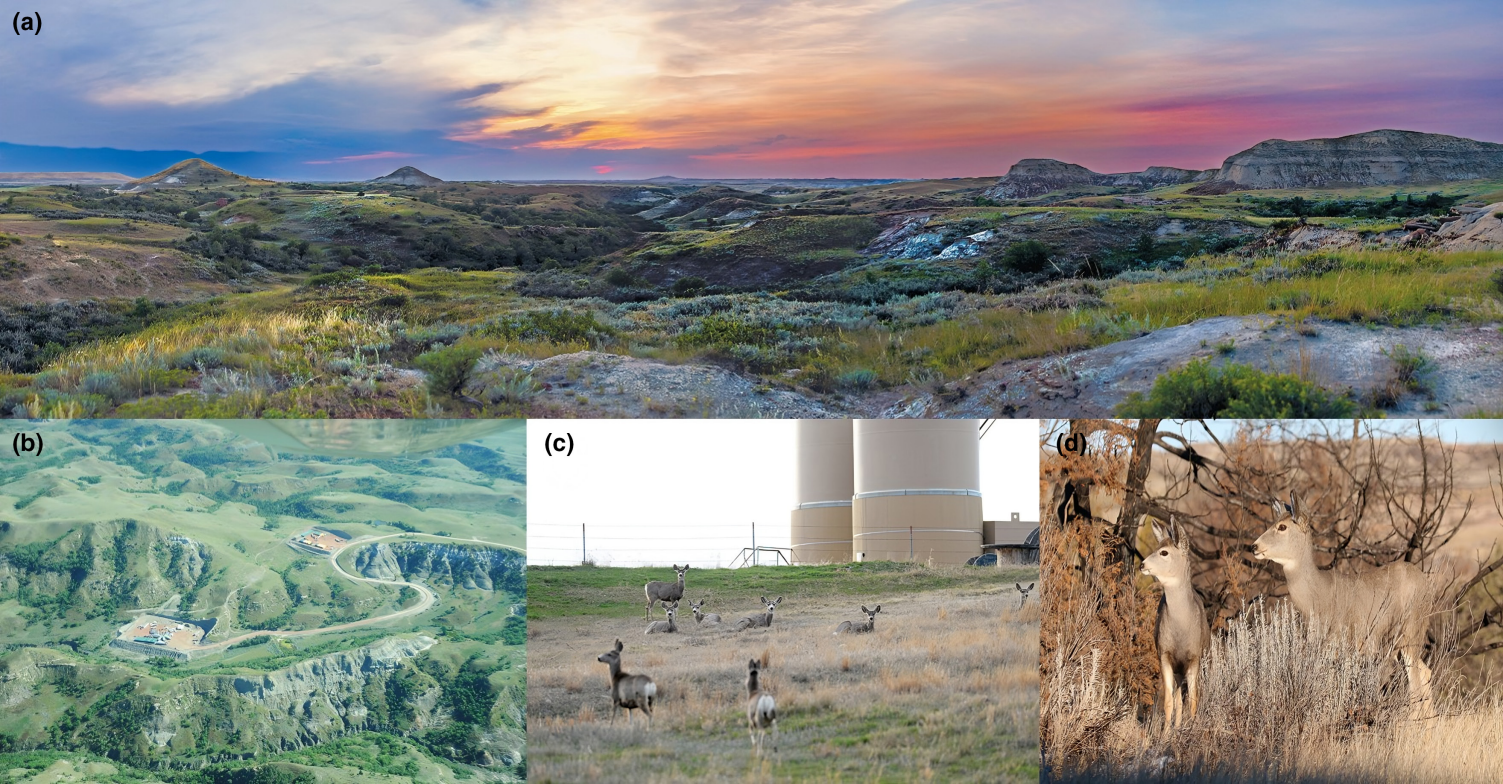
Selected photos from our study area depicting (a) typical landscape features of the North Dakota badlands, (b) an operational well pad, (c) mule deer in proximity to an industrial facility, and (d) a mule deer doe with fawn. Photos: Ashley Salwey, Jesse Kolar and Craig Bihrle, North Dakota Game and Fish.

**FIGURE 2:**
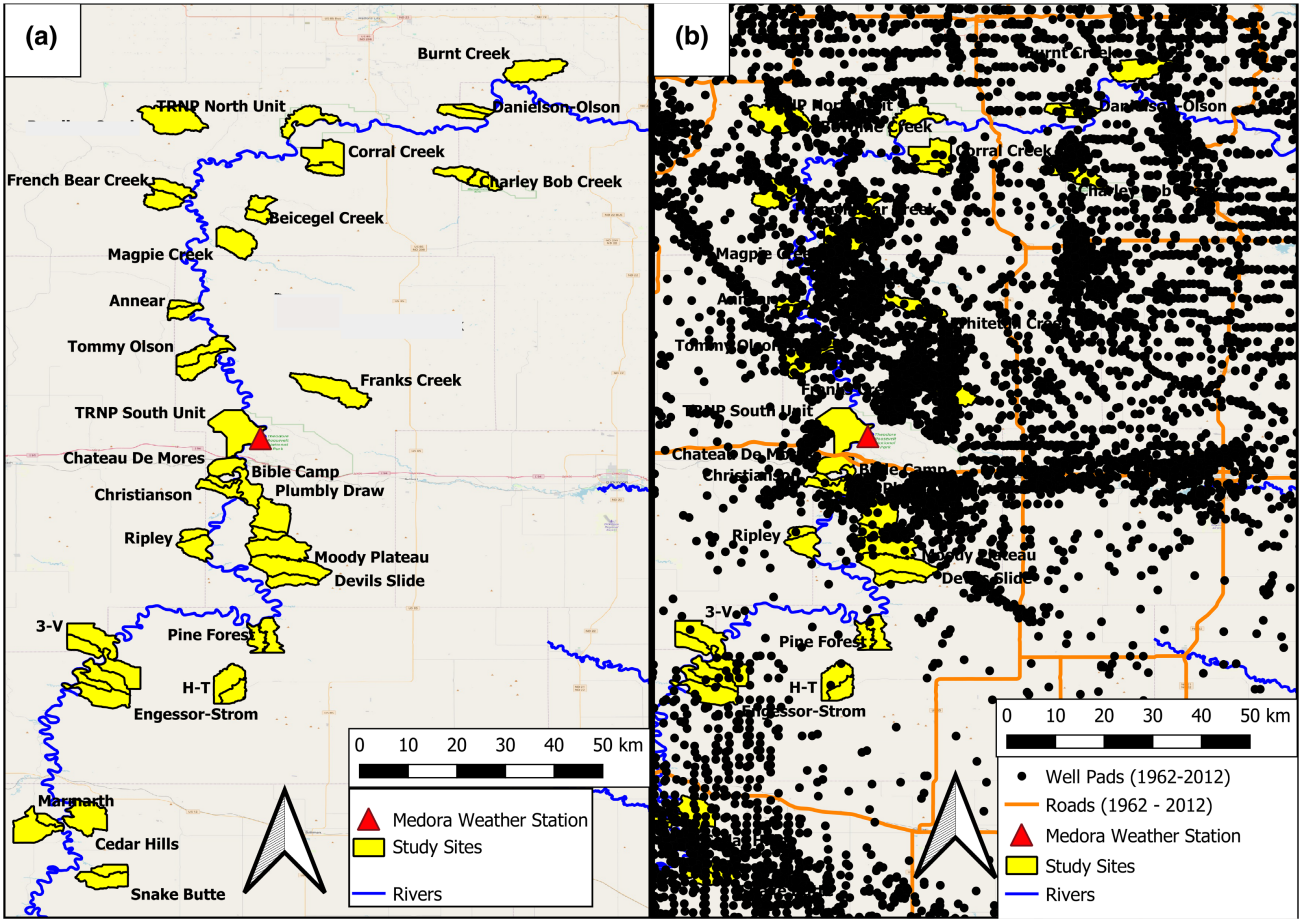
Map of our study area in North Dakota, showing (a) the location of mule deer study sites (in yellow) in North Dakota in relation to the Little Missouri river and the location of the Medora weather station (red triangle, middle of image, and bordering river) and (b) the total cumulative location of individual active well sites between 1962 and 2012 (black dots) and major highways (orange network) in relation to our study sites.

Study sites were located in the Little Missouri Badlands, the primary range of mule deer populations in southwestern North Dakota, USA. The Badlands are typified by steep slopes (615–913 m above sea level), minimal vegetation productivity, and extensive drainage systems due to erosion (Figure 1a). We considered historical mule deer aerial surveys gathered from 26 study sites located along the Little Missouri River (Figure 2a). While Rocky Mountain Juniper (*Juniperus scopulorum*) is the prevailing woody plant on north‐facing slopes in the Badlands, wooded draws of green ash (*Fraxinus pennsylvanica*) dissect much of the area and with shallower slopes containing a mix of shrublands (sagebrush *Artemisia* spp.), buffaloberry (*Shepherdia argentea*), skunkbrush sumac (*Rhus aromatica*), greasewood (*Sarcobatus vericulatus*), rabbitbrush (*Chrysothamnus nauseosus*), and native mixed grass prairies. North Dakota's climate is continental, and temperatures are highly variable both seasonally and daily. Temperatures range from a mean daily high of 30.8°C in July (1962–2012: range 23.5–34.5°C) to a mean daily low of −16.1°C in January (range −24.9 to −6.5°C). Snow cover typically occurs between November and April. Mule deer share this landscape with low‐density populations of pronghorn (*Antilocapra americana*), white‐tailed deer *(Odocoileus virginianus*), elk (*Cervus elaphus*), and bighorn sheep (*Ovis canadensis*). Coyotes (*Canis latrans*) are the primary predator of mule deer and their fawns in our study sites (Jensen, 1988).

### 2.2 Mule deer aerial surveys in fall (fawn fall recruitment) and spring (density)

**FIGURE 3:**
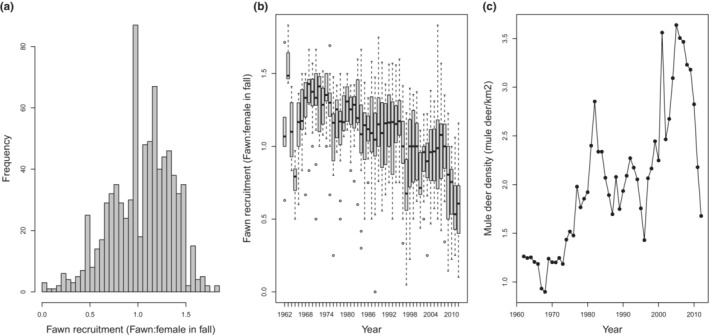
Long‐term population metrics for mule deer over the study period (1962–2012). Mule deer fawn recruitment, calculated as the ratio of fawns: female in fall (a), boxplots representing the variability among the 26 study sites (b) and mule deer density calculated as the total number of animals in spring (c) across 26 study sites in south‐west North Dakota.

Between 1962 and 2012, the North Dakota Fish and Game Department surveyed small blocks (Figure 2a) to assess mule deer population parameters (counts categorized as males, females, and fawns) each October after tree leaf‐fall and before the deer rifle hunting season. Mule deer were surveyed from fixed‐wing aircraft. Airplanes were flown at as low an altitude (76–106 m) and airspeed as possible (<129 km/h) for 100% coverage of a survey block. Flight paths within study sites were standardized and replicated year after year. Recruitment was then recorded as the fawn:female ratio observed per study site and year, note that values >1 indicate multiple fawns per female (Figure 3, panel a,b). The observer during the flight responsible for spotting the deer was also recorded. Through the 50‐year monitoring period, the same sites were surveyed again in April after snowmelt and before trees leafed out. Spring surveys were performed to estimate mule deer density across the 26 study sites (Figure 3: panel c).

**FIGURE 4:**
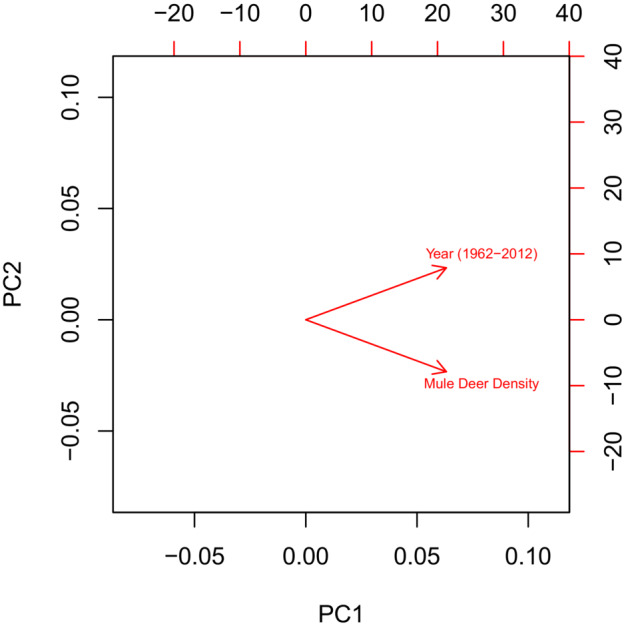
Biplot for the principal component analysis for year of study and mule deer density. When PC1 values increase, mule deer density increases over time, whereas when PC2 values increase mule density declines along the time series 1962–2012. Including PC1 and PC2 in our recruitment model allows us to distinguish between density‐dependent effects (PC1, 87.9% variability explained) and recruitment decline due to other factors (PC2, 12.1% variability explained).

Year and mule deer density were collinear in this dataset (Pearson's correlation coefficient *r*_*p*_ = .75, Figure 3b). Increasing deer densities over time combined with a steady decrease of fawn recruitment along the time series (Figure 3a) suggested a density‐dependent effect in the preliminary stage of our analysis. The collinearity between year and mule deer density prohibited running models with both predictors (Dormann et al., 2013). However, it was important to include the time‐series (year) in the model to reduce residual temporal autocorrelation and mule deer density to assess the long‐term effect of density‐dependent effects on recruitment. Therefore, we ran a principal component analysis using the *prcomp* function within the *stats* package (R Development Core Team, 2021) to include information from both predictors. We included both principal components which accounted for 100% of the variability of both predictors. PC1 was included to capture density‐dependent effects (87.9% variability explained, Figure 4). PC1 increases in value as mule deer density increases over time, whereas PC2 encapsulated years where mule deer density decreased over time (12.1% variability explained, Figure 4). We used the principal components of year and mule deer density to explain variation in recruitment that is not explained by density‐dependent effects over time.

### 2.3 Weather data

We split the year into 3 main seasons where weather is expected to drive fawn recruitment. We expect that unfavorable weather conditions recorded prior to birth (winter: November–March), during the birth season (spring: April–May) and immediately after the birth (summer: June–September) of fawns negatively affect fawn survival and, consequently, fawn recruitment recorded during the following October. Harsh winter conditions were expected to weaken female body condition with consequences on their reproductive success (Ciuti et al., 2015; Forrester & Wittmer, 2013). At the same time, snowy and cold spring weather can debilitate female body condition after fat reserves in females were depleted over winter (Forrester & Wittmer, 2013). Unfavorable spring weather extends winter, with snow sometimes persisting for longer periods and delaying green‐up (Ciuti et al., 2015). Finally, hot and dry summers are expected to reduce food quality and availability thus reducing fawn survival (Jensen, 1988). We extracted annual values of average minimum temperature (°C), average precipitation (mm), and average snow depth (cm) data for winter (mean minimum temperature: −11.2°C; mean precipitation: 12.71 mm; mean snow depth: 63.76 mm) and spring (mean minimum temperature: 2.1°C; mean precipitation: 51.17 mm; mean snow depth: 5.49 mm), and average minimum temperature (°C) and average precipitation (mm) for summer (mean minimum temperature: 10.4°C; mean precipitation: 48.69 mm) from the Medora weather station (station id: Medora ND US; elevation 686.1 m a.s.l.; Latitude 46.966° N; Longitude −103.500° W) centrally located within the mule deer range in our study area (Figure 2). This station has been shown to provide reliable weather data for the region previously used in mule deer population modeling (Ciuti et al., 2015).

### 2.4 Landscape data

Our study sites consist of varying landscape features derived from geological history of the region. The Little Missouri River flowing north was caused by glaciers depressing the land. As the glaciers melted, the land experienced isostatic rebound, with erosion most severe in the northern Badlands. The northern Badlands, having an older history of erosion, are characterized by steep‐sided washes and dry creeks. These shaded steeper slopes support more Rocky Mountain juniper trees and are the primary range of mule deer in this region. The middle Badlands have less steep terrain and support deciduous woody draws and Rocky Mountain juniper on north facing slopes. The southern Badlands are the least dissected by washes, consisting primarily of short grass prairies broken by smaller drainages, with more undulating terrain, and fewer trees. Over the course of our study, encroachment of Rocky Mountain juniper has occurred in areas across the region, thus altering the availability of quality habitats for mule deer.

To describe the heterogeneity of the environment across the 26 study sites spread along the Little Missouri River (Figure 2a), we considered the following variables at the study site level: elevation and slope variances to capture the different erosion processes and related landscape configuration (static variables, being unique values at the study site level across the 50 years of the time series); woody vegetation encroachment (percentage cover), a dynamic variable able to embrace the level of Rocky Mountain juniper encroachment by study site and year; and, finally, latitude and longitude, which are expected to describe the remaining variability in landscape variability. Woody vegetation encroachment was obtained by a previous modeling effort (Ciuti et al., 2014), This was obtained by digitizing 54 random plots for which replicated aerial photos from 1939 to 2012 were available. For each plot, woody vegetation coverage was digitized, and encroachment rate was modeled using linear regression and a set of candidate predictors (latitude, longitude, aspect, habitat class, geological features, and ruggedness). Starting from a full model, the best linear model was selected using backward stepwise model selection; the best model explained 40% of the variability in woody vegetation covering mule deer study sites. This model was used to rebuild the degree of yearly encroachment per study site (Ciuti et al., 2014).

**FIGURE 5:**
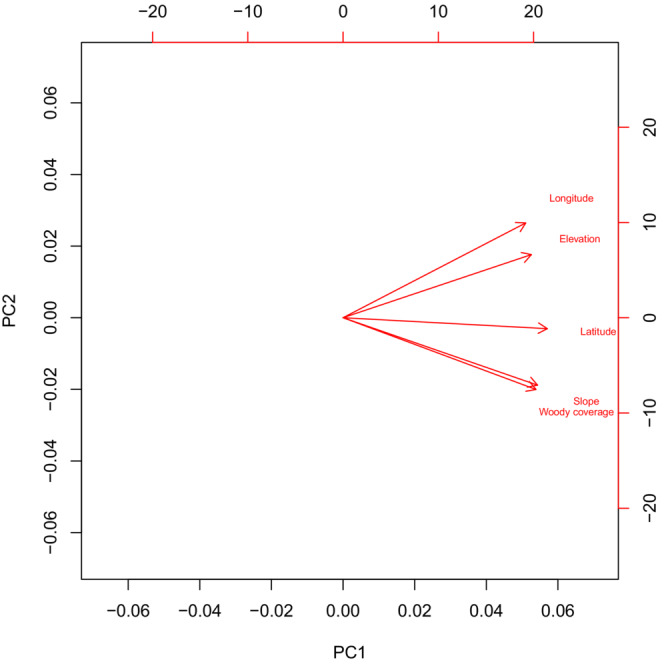
Biplot for the principle component analysis of landscape predictors included in our models. Increasing values of PC1 represents study sites in the north‐east that have experienced encroachment of Rocky Mountain juniper, whereas increasing values of PC2 accounts for study sites in the north‐east that have rugged terrain and less encroachment of woody vegetation, PC3 is shown in Appendix S1: supplementary material 1.

The five spatially explicit variables described above were highly correlated (e.g., rougher and more encroached landscape when moving from south‐west toward north‐east). We conducted a principal component analysis using the *prcomp* function within the *stats* package (R Development Core Team, 2021) and retained the first 3 of the 5 principal components accounting for 93.94% of the variability (variability explained by PC1: 79.0%; PC2: 9.7%, and PC3: 5.1%; Figure 5, Appendix S1: Supplementary Material 1). Whereas increasing values of PC1 correspond to generally steeper, more juniper encroached study areas moving along the Little Missouri River toward North‐East, increasing values of PC2 correspond to eastern study sites which have been less affected by Rocky Mountain juniper encroachment.

### 2.5 Disturbance data

**FIGURE 6:**
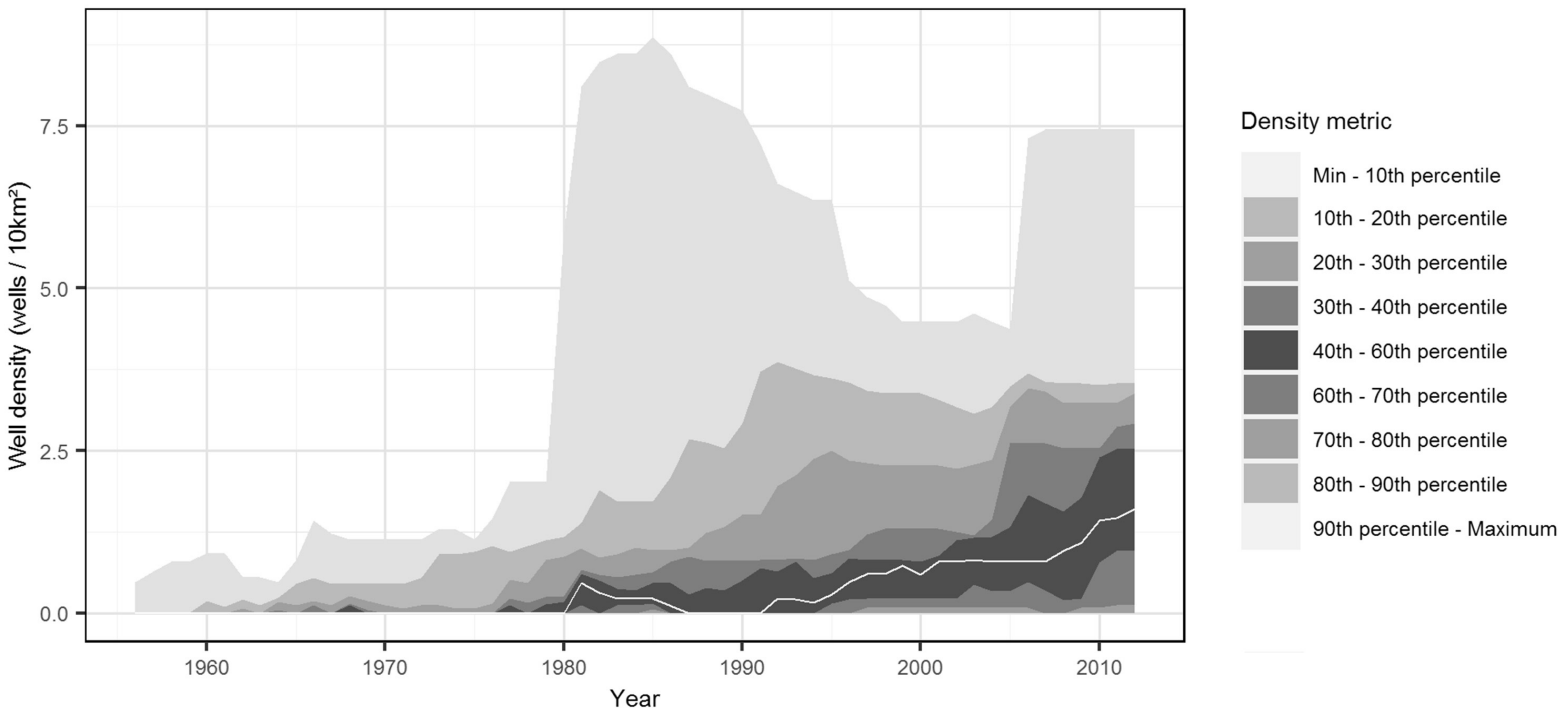
Fan chart of percentiles of oil and gas active well density (in shaded 10‐percentile intervals) across 26 study areas in the North Dakota badlands for the years of record (1962–2012). The median density is shown as a white line.

The density and activity of active oil and gas wells (active wells/10 km^2^) inside mule deer survey units and within a buffer of 1 km was computed with data from satellite imagery, governmental data, and information from extraction companies for the period 1962–2012 (Figures 2b and 6). Contrary to usual energy development maps that are static over time (i.e., location of the wells drilled across a given study period), we rebuilt the life history of every well drilled from 1962 to 2012 in the mule deer study sites (Figures 2b and 6). In our final database, we associated the density of active wells in a given year to the corresponding mule deer study site. If wells were abandoned over time (inactive), then they were not considered in the well density estimates because we assumed the level of disturbance to wildlife is not obtrusive for inactive wells. We also included road density in our models (computed as the kilometers of roads per km^2^). This was calculated for each year in our time series (1962–2012) as the km of roads per km^2^ of the study area to account for annual variation in road development. Annual road density in our study area was positively correlated (but not collinear) with annual active well density (Pearson's correlation coefficient *r*_*p*_ = .19).

### 2.6 Predator data

Coyote observations were collected during mule deer aerial surveys in spring as incidental observations. These coyote observations are likely the least robust data in our dataset, being derived from opportunistic observations during mule deer aerial surveys. That said, these data are unique for such a long time series, and we carefully evaluated the data prior to including it in our recruitment model. During preliminary analysis, we considered using a relative density estimate computed as coyotes per study site; however, these did not provide robust estimates due to limited sample sizes per study site compared to the whole region. Rather, we calculated overall coyote density for the 26 study areas (hereafter also referred to as coyote density) over the course of the study period to have a consistent index of annual coyote density in spring. We used these data with the assumption that such estimates underestimate the actual coyote density but provide a long‐term repeatedly collected index of interannual variation in predator presence and pressure on mule deer populations across the study area. Our coyote density estimates (1962–2012) were significantly positively correlated (Pearson's correlation coefficient *r*_*p*_ = .25) with another independent index of coyote density across the Badlands derived from incidental observations by rural mail carriers (available from 1980 to 2012).

### 2.7 Generalized additive mixed‐effect models

We modeled the data using generalized additive mixed‐effect models (Wood, 2017) using the *gam* function from the *mgcv* package in R (Wood, 2017) to examine the relationship between mule deer fawn recruitment and our selected covariates. Our final database contained 809 observations across 26 study sites and a 50‐year time series. Our basic model structure took the form

$$\begin{array}{c} \text{Fawn}:\text{female ratio}∼\text{intercept}\\ +f_{1}\left(\chi_{1}\right)+\ldots +f_{13}\left(\chi_{13}\right)+f_{14}\left(\text{Observer}\right)+f_{15}\left(\text{StudyArea}\right)+\text{error}\;\left(\text{Gaussian}\right) \end{array}$$

where the response variable is fawn:female ratio in fall and each covariate *f*_1_($\chi_{1}$)… *f*_13_($\chi_{13}$) in the alternative model structure (explained below) is fit with a smooth term function defined by *f*_1_ to *f*_13_ to allow for nonlinear effects. The smooth term functions included in the model are composed of sections of cubic polynomials, which are joined together at specified locations known as *knots* (controlled by a value *K* in the model). We conservatively set the *K* value for each covariate in the model to define the number of knots in each covariate spline, thus allowing the model to learn from the data and penalize the regression spline accordingly to avoid overfitting the data (Wood, 2006). We also included *Observer and StudyArea* as random effects. We opted to use a Gaussian distribution of errors (identity link) that met the assumptions of homogeneity and normality (Wood, 2017). We screened the predictor data to check for gaps in the data space coverage, which highlighted a number of variables that needed to be log10 transformed (i.e., spring precipitation, spring mule deer density, coyote density, road density, and well density). Gaps in the data space coverage can bias model estimates in generalized additive models, and thus, we followed the step‐by‐step protocol defined by Zuur et al. (2009), which recommends log10 transforming data to ensure good coverage. We fit alternative a priori models including one seasonal weather predictor per model (i.e., one of—average minimum temperature, average precipitation and where relevant, average snow depth—for winter, spring, and summer, respectively). Thus, we had 3 weather predictors for winter and spring and 2 for summer (where snow depth is irrelevant), resulting in a final set of 18 unique alternative models (3 winter variables x 3 spring variables x 2 summer variables). We used second‐order Akaike Information Criterion (AICc) to rank alternative models and the null model. AICc values for each of the models were extracted from the *Mumin* package in R (Barton, 2022); however, we did not use the *Mumin* package to rank the models, rather we followed classical approach to theoretic information criterion model ranking (Burnham et al., 2011).

Each model had the same a priori structure with the exception of alternative weather predictors. We designed this structure based on our understanding of the local animal ecology to examine the effects of weather, disturbances, and predators on mule deer recruitment over the 50‐year period. Specifically, we modeled fawn:female ratio as a function of single terms—two principal components computed using year and mule deer density data, three principal components computed using landscape data (elevation variance, slope variance, woody vegetation percentage, longitude, and latitude), annual road density, and spring mule deer density as smoothing splines. We included interactions for seasonal weather and coyote density—to test whether predator pressure had a differential effect depending on weather harshness—and seasonal weather and well density—to test whether energy development interacted with weather harshness in affecting fawn recruitment. Finally, we included an interaction between well density and coyote density to test our hypothesis of the differential ability of coyotes to prey upon fawns based on energy development‐related habitat modification and loss. We also included crossed random intercepts for the observer on the plane and survey block (ID). The models were fit using the select = TRUE implementation in the *gam* algorithm of the *mgcv* package: this option adds a penalty to each smoothing term, allowing it to be penalized out of the model (when judged irrelevant by the algorithm) via optimization of the smoothing parameter selection criterion (Wood, 2017). Normality and homogeneity of GAMM residuals were successfully met by inspecting quantile‐quantile (QQ) plots and simulated residual plots from the *DHARMa* package in R (Hartig, 2021). We examined the model residuals for spatial (*R package: sp*; *function: variogram*) and temporal (*R package: stats*; *function: acf*) autocorrelation (Cressie, 1993; Venables & Ripley, 2002).

## 3 RESULTS

Of the 18 a priori generalized additive mixed‐effect models fitted and compared with AICc, we selected the top‐ranked model (pseudo‐*R* ^2^ = 0.452; Deviance Explained = 47.8%, Table 1) containing average minimum temperature in winter, average snow depth in spring, and average minimum temperature in summer as proxies for seasonal weather. The second‐ranked model was 1.18 AICc points lower than the top‐ranked model (differing from the top‐model only by having minimum temperature instead of snow depth as a spring weather proxy; we present the effects of spring minimum temperature in Appendix S1: Supplementary Material 2). Note that this modeling strategy has been adopted to identify the best weather indices, which were suggested by the lowest AICc value of the top‐ranked model. We were not interested in model comparisons and averaging because structure was identical among models with the only exception being the included proxies for seasonal weather. We report approximate significance of smooth terms (including crossed random intercepts for survey observer and study area) for the top‐ranked GAMM in Table 2. We did not find evidence of temporal (Appendix S1: Supplementary Material 3) or spatial (Appendix S1: Supplementary Material 4) autocorrelation in the residuals of the top‐ranked model.

**TABLE 1:** Alternative generalized additive mixed‐effect models explaining the variability of mule deer recruitment in the North Dakota badlands from 1962 to 2012.

Winter	Spring	Summer	LogLik	AICc	ΔAICc
Minimum temperature	Snow depth	Minimum temperature	37.93	4.12	0
Minimum temperature	Minimum temperature	Minimum temperature	32.8	5.30	1.18
Minimum temperature	Precipitation	Minimum temperature	30.96	7.29	3.16
Minimum temperature	Snow depth	Precipitation	30.84	11.82	7.7
Snow depth	Minimum temperature	Minimum temperature	28.45	16.73	12.6
Minimum temperature	Precipitation	Precipitation	21.97	24.31	20.19
Minimum temperature	Minimum Temperature	Precipitation	23.6	24.62	20.5
Precipitation	Snow depth	Minimum temperature	23.91	27.93	23.8
Snow depth	Snow depth	Minimum temperature	20.08	29.57	25.45
Snow depth	Precipitation	Minimum temperature	14.94	34.04	29.92
Snow depth	Minimum temperature	Precipitation	14.69	36.78	32.65
Snow depth	Snow depth	Precipitation	18.68	38.26	34.14
Precipitation	Minimum temperature	Minimum temperature	18.73	43.86	39.74
Snow depth	Precipitation	Precipitation	5.58	46.32	42.2
Precipitation	Precipitation	Minimum temperature	14.29	48.14	44.01
Precipitation	Precipitation	Precipitation	4.8	53.76	49.64
Precipitation	Snow depth	Precipitation	7.15	55.65	51.53
Precipitation	Minimum temperature	Precipitation	−1.18	63.04	58.92

*Note*: Models have been ranked using the corrected Akaike Information Criterion AICc (LogLik: log‐likelihood; ΔAICc: difference in AIC between any model and the top ranked one). All models had the same a priori structure (see text for full details), except for different combinations of the average seasonal weather proxies in the 3 main seasons of the mule deer annual biological cycle.

**TABLE 2:** Approximate significance of smooth terms as estimated by our top generalized additive mixed‐effect model explaining the variability of mule deer recruitment in the North Dakota Badlands from 1962 to 2012.

Smooth term	Edf	Ref.df	*F* statistic	*p* value
*f*_1_ (PC1_Year,Density_)	0.09	4	11.728	.001
*f*_2_ (PC2_Year,Density_)	0.08	4	11.09	.001
*f*_3_ (PC1_Landscape_)	0.000035	4	0	.532
*f*_4_ (PC2_Landscape_)	0.96	4	6.242	.001
*f*_5_ (PC3_Landscape_)	0.37	4	0.141	.328
*f*_6_ (Road density)	0.22	4	0.078	.296
*f*_7_ (Well density, Winter temperature)	1.99	7	6.106	<.001
*f*_8_ (Well density, Spring snow depth)	3.70	6	2.276	.010
*f*_9_ (Well density, Summer temperature)	0.15	6	0.035	.237
*f*_10_ (Coyote density, Winter temperature)	5.69	6	5.447	<.001
*f*_11_ (Coyote density, Spring snow depth)	0.000066	5	0	.035
*f*_12_ (Coyote density, Summer temperature)	3.51	5	8.723	<.001
*f*_13_ (Coyote density, Well density)	0.00	5	0	.765
*f*_14_ (Observer)	14.30	22	5.919	<.001
*f*_15_ (Study area)	5.29	25	0.3	.177

*Note*: Note that survey observer and study area have been fitted as crossed random intercepts. Smooth terms containing two explanatory variables represent interactions in the model.

**FIGURE 7:**
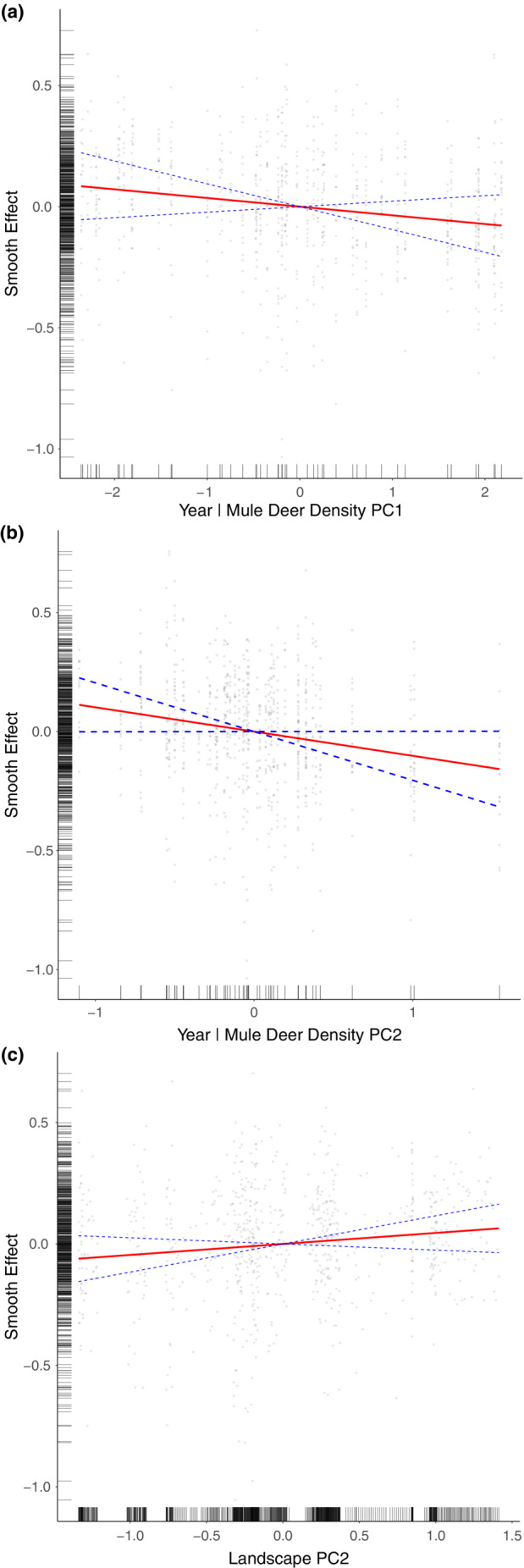
Effect of year and mule deer density principal components on mule deer fawn recruitment. PC1 increases (and recruitment decreases) when mule deer density increases with year of the study (a). PC2 increases (and recruitment decreases accordingly) when mule deer density decreases, accounting for years of decline not attributable to density‐dependent effects (b) and the effect of landscape increasing values of PC2 on mule deer fawn recruitment, where recruitment increases in their core range (north‐east) that is unaffected by the encroachment of woody vegetation such as Rocky Mountain juniper (c).

For the top‐ranked model (Table 2; see Appendix S1: Supplementary Material 5 for the smoothing splines not included as figures here), we found a negative relationship between PC1 computed using year and mule deer density, denoting a density‐dependent effect (Table 2; Figure 7a). Likewise, we found PC2 for year and mule deer density to be negatively related to recruitment, this time explaining the decline in recruitment over time that was not due to density‐dependent effects (Table 2; Figure 7b).

We found no effect of PC1 computed using landscape data in the model (Table 2; Appendix S1: Supplementary Material 5A). We did find a significant effect of PC2 on recruitment (Table 2, Figure 7c); this describes an increase in recruitment in the north‐eastern survey blocks less affected by woody vegetation encroachment. Finally, PC3 provided no effect on mule deer fawn recruitment (Table 2; Appendix S1: Supplementary Material 5B). Road density also had no effect on mule deer fawn recruitment (Table 2; Appendix S1: Supplementary Material 5C).

**FIGURE 8:**
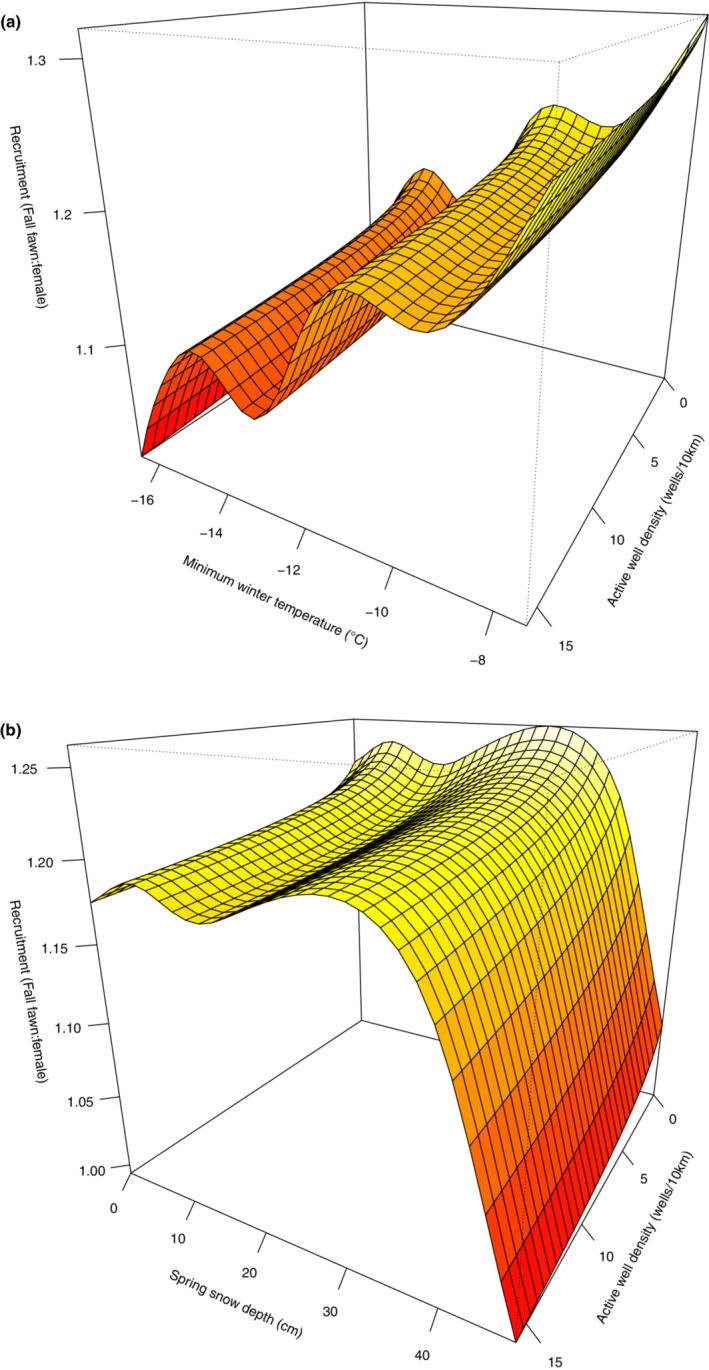
Interactive effects of active well density and average winter minimum temperature (a) and active well density and average snow depth in spring (b) on fall mule deer fawn recruitment (fawn/female ratio) in the North Dakota badlands (1962–2012) as predicted by the top ranked generalized additive mixed effect model.

Active well density had a subtle but significant interaction with winter and spring seasonal weather in finely modulating mule deer recruitment. We found that active well density interacted with average minimum winter temperature (Table 2; Figure 8a), when we found higher recruitment when active wells occurred at lower densities and winters were milder (Figure 8a). Similarly, we found a significant interaction between active well density and snow depth in spring (Table 2; Figure 8b), with snow depth strongly and inversely correlated to recruitment (particularly when exceeding ~25–30 cm of snow on the ground) while being slightly negatively impacted by active well density (Figure 8b). We found no significant effect of active well density interacting with average minimum summer temperature (Table 2, Appendix S1: Supplementary Material 5D).

**FIGURE 9:**
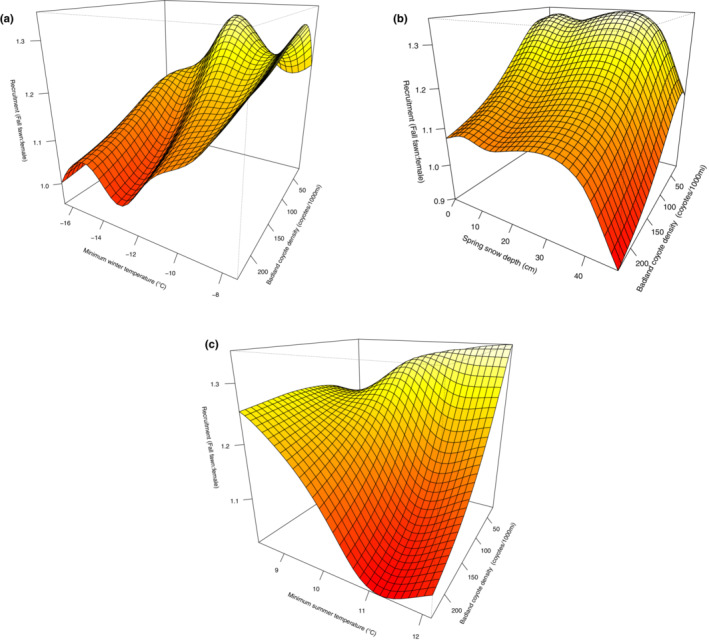
Interactive effects of coyote density and average winter minimum temperature (a), coyote density and average snow depth in spring (b), and coyote density and average summer minimum temperature (c) on fall mule deer fawn recruitment (fawn/female ratio) in the North Dakota badlands (1962–2012) as predicted by the top ranked generalized additive mixed effect model.

We found an interaction between coyote density and winter harshness (Table 2; Figure 9a). Fawn recruitment was higher during milder winters and when coyotes occurred at lower densities, the opposite effect on recruitment being recorded during colder winters and higher coyote densities (Figure 9a). Similarly, higher coyote densities and deeper snow depth in spring had a negative interactive effect on mule deer recruitment (Table 2, Figure 9b). We also found that hot summer temperatures, in combination with higher coyote density, led to a reduction in recruitment (Table 2, Figure 9c), with high predator density particularly impacting recruitment during hot summers.

**FIGURE 10:**
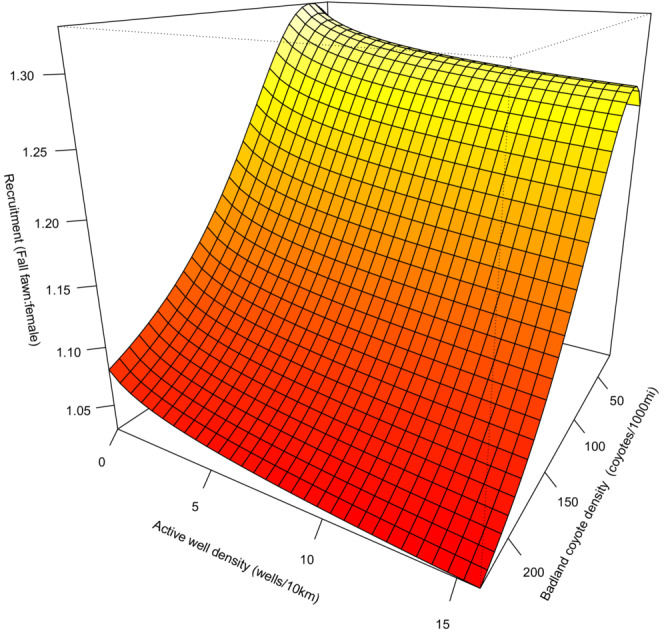
Interactive effect of coyote density and well density on fall mule deer fawn recruitment (fawn/female ratio) in the North Dakota badlands (1962–2012) as predicted by the top ranked generalized additive mixed effect model. Note that this interaction was not significant in the top‐ranked model.

Finally, we did not find a significant interaction between coyote density and active well density in affecting mule deer recruitment (Table 2; Figure 10). Our data suggests that coyote density and active well density are not interactive factors in this predator–prey system. We indeed found a weak but significant negative effect of well density on fawn recruitment irrespective of coyote density. Likewise, we found a stronger negative effect of coyote density on fawn recruitment irrespective of active well density (Figure 10). Thus, the effects of predator density and energy development appear to be additive (rather than interactive) in shaping mule deer fawn recruitment fluctuations in the North Dakota Badlands. We have included all interaction plots as single panel plots, with 95% confidence intervals shown in Appendix S1: Figure S1.

## 4 DISCUSSION

During the late 1800s, mule deer populations, like other big game species in the western United States, were severely reduced by over exploitation from unregulated hunting. Following the establishment of regulated hunting seasons, mule deer populations increased rapidly and peaked in the 1960s (Knue, 1991). In the early to mid‐1970s, mule deer numbers began to decline again (Jensen et al., 2023). Biologistshave attributed these declines to a variety of factors across the United States and Canada ranging from habitat loss and degradation (AB, CO, ID, NV, OR, WA, and WY), severe winter weather (AB, BC, CA, CO, MT, SK, and UT), drought (AZ, NM, and UT), reduced forage quality and quantity (CA, CO, and NM), hunting (TX and UT), predation (BC and CA), and domestic livestock (CO) (Doyle et al., 1993; Ziegler & Myers, 1983). In contrast, black‐tailed deer (*O. h. columbianus and O. h. sitkensis*) populations seemed less affected by these factors (Gill, 1990). Understanding the primary factors driving mule deer and other ungulate populations is essential for developing informed and effective management strategies (Forrester & Wittmer, 2013; Peek et al., 2002; Sawyer et al., 2017). Our study was motivated by the need to understand if landscape‐level human disturbance from energy development over a 50‐year period (1962–2012) had influenced mule deer recruitment in the North Dakota Badlands. With the North Dakota Badlands being on the eastern periphery of mule deer range distributions, we predicted oil and gas development might have a strong influence on fawn recruitment. This was not the case, rather, we found that mule deer recruitment trends were largely driven by biotic (population dynamics, habitat quality) and abiotic factors (seasonal weather severity and landscape variability) with human disturbance from oil and gas development and predation seeming to play a subtle and additive role in influencing annual oscillations in fawn recruitment.

The strong influence of density dependence on fall fawn recruitment over time suggests limited quality habitat to be the primary driver of mule deer in this northern Great Plains population, with Rocky Mountain juniper encroachment being a key factor of habitat degradation for mule deer. Density dependence is further supported by the observation that within Theodore Roosevelt National Park, a nonhunted population with a mule deer density 69% higher than outside the park, had a fall fawn recruitment rate that was 24% lower than through the Badlands (Jensen et al., 2022). Density‐dependent effects in wildlife are manifested in a series of feedback mechanisms that lead to fluctuations in populations depending on the relative density of the animal. Density‐dependent effects are well studied in wildlife (Hixon & Webster, 2002; Tanner, 1966) and, in particular, in wild ungulate populations (Brown, 2011; Gaillard et al., 1998; Sæther, 1997). Here we found another example of density‐dependent effects in North Dakota mule deer, where the effect likely modulates populations through intraspecific competition for high‐quality habitats. We also observed greater declines in recruitment in areas where primary mule deer habitat has been affected by encroachment of juniper. Mule deer are selective foragers and body condition is closely related to the quality of available resources, in contrast to high quantities of less nutritious foods (Wallmo, 1981). Mule deer have been observed to adjust their dietary niche when populations occur at higher densities with evidence of niche expansion (Stewart et al., 2011) and contraction (Nicholson et al., 2006), resulting in negative consequences for population productivity. Deer use fat and protein reserves accumulated during late summer and fall to survive the winter. As a result, behavioral changes due to high population density, or trade‐offs due to perceived predation risk, have fitness and reproductive consequences due to suboptimal body condition. Effects of reduced body condition are exacerbated by harsh seasonal weather, habitat integrity, and intraspecific competition for high‐quality forage (Christie et al., 2015; Ciuti et al., 2015; Gamo & Beck, 2017).

Following density dependence, stochastic severe winter weather, particularly cold temperatures, and snow depths >25 cm were found to have secondary impacts on recruitment of fawns to the following fall. Harsh winter weather is known to weaken female body condition and decreases reproductive success, and unfavorable spring weather prolongs negative impacts of winter conditions, for instance, when snow persists and delays green‐up of foraging sites (Ciuti et al., 2015; Parker et al., 1996; Torbit, Carpenter, Alldredge, et al., 1985; Torbit, Carpenter, Swift, et al., 1985). Whereas, hot and dry summers were expected to reduce food quality and availability, resulting in reduced fawn survival and subsequently lower autumn recruitment (Ciuti et al., 2015; Hurley et al., 2011). Seasonal weather affects energy expenditure (Freddy et al., 1986) and forage quality (Laycoek & Price, 1970 ) and subsequently body condition (Torbit, Carpenter, Alldredge, et al., 1985; Torbit, Carpenter, Swift, et al., 1985). Hot summers and higher coyote densities were found to accompany lower fawn recruitment. Drought conditions could have resulted in poor body condition and less vegetative concealment cover for fawns and thus increased fawn susceptibility to predation (Ballard et al., 2001).

The effects of energy development and predation, and their relative roles in the ecology of mule deer in North Dakota may not have been detected with a shorter‐term study, although, Kolar et al. (2017) estimated a 24% decline in survival when well‐pad density increases from 0 to 1.93 well pads/km^2^. This coincides with levels and thresholds of high development in the guidelines established by the Western Association of Fish and Wildlife Agencies (Lutz et al., 2011). Terrain may have played a role in other studies that have documented more profound effects of energy development. For example, in the relative flat and open terrain of western Wyoming, Sawyer et al. (2017) documented substantial impacts of energy development on mule deer. In contrast, the rougher and highly dissected terrain of the North Dakota Badlands could play a role in mitigating the response of mule deer to human disturbance. Gullikson (2019) reported avoidance of oil wells by white‐tailed deer (*Odocoileus virginianus*) in western North Dakota, but physical barriers or suitable concealment cover habitat adjacent to well pads can allow deer to tolerate wells and associated activity.

Despite predators, coyotes in our study, being a primary source of direct mortality for both adults and juveniles (Forrester & Wittmer, 2013; Lingle et al., 2008), it is rare that predation causes long‐term population declines in mule deer (Forrester & Wittmer, 2013). A review of deer–predator relationships (Ballard et al., 2001) reported that coyotes, mountain lions (*Puma concolor*), and wolves might be substantial mortality factors in some areas under certain conditions, but large herbivores at, or near, carrying capacity do not exhibit strong responses to predator removal, as emphasized recently by Bowyer et al. (2005, 2014, 2020). In our study, in years where we recorded harsh seasonal weather conditions, coyotes had a measurable effect on reducing fall mule deer recruitment. Coyote‐induced mortality to mule deer populations is typically compensatory when prey density is high (i.e., when predator‐induced mortality increases, other forms of mortality typically decrease, Bergman et al., 2015), therefore it will not reduce populations. Coyote predation can be additive (i.e., capable of limiting recruitment supplementary to other forms of mortality) if other sources of proximate mortality are higher, such as weather severity (Bergman et al., 2015). In addition to direct predation of mule deer fawns, coyotes may also influence mule deer behavior and space use through nonconsumptive effects (Laundré et al., 2010), also called a trait‐mediated effect which cause behavioral changes, often with fitness consequences, as prey balance the need for high‐quality forage, protective habitat, and safety from perceived predation risk (Frid & Dill, 2002; Preisser & Bolnick, 2008; Walther, 1969). We acknowledge that the data collected on coyotes for this study were incidental observations during mule deer surveys: that said, these data provide a good proxy for overall coyote trends over the course of this 50‐year study; however, they likely do not accurately quantify the exact impact of predation on mule deer recruitment across a larger region. We therefore recommended that a targeted data collection of coyote population trends across mule deer jurisdictional ranges needed to quantify the degree to which coyote predation causes additive mortality for mule deer fawns.

Our data suggest that human disturbance might play a similar role in modulating mule deer fitness if the placement of oil and gas wells alters mule deer habitats and behavior. We observed, for the first time using high‐quality spatial data depicting the life‐history of all wells in the region over 50 years, that there was an effect of active well density which was significant when interacting with average minimum winter temperature and with average snow depth in spring. Human disturbance and the modification of ecosystems with human infrastructure can change prey behavior, as it is possible for prey to respond to human activity in the same way they respond to predators (Ellrich et al., 2015; Frid & Dill, 2002; Kolar et al., 2017; Sawyer et al., 2017). This phenomenon has been observed in many marine and terrestrial species displaying avoidance of human presence and related infrastructures (Allen & Read, 2000; de la Torre et al., 2000; Dyer et al., 2001) as well as in mule deer avoiding energy development sites across their range (Kolar et al., 2017; Lynch et al., 2015; Sawyer et al., 2017). Thus, in years where winter weather is harsh and persists into spring, the effect of landscape modification from energy development appears to impact mule deer fitness but does not alter predator–prey relationships. Contrary to our expectations, and as suggested by earlier analysis (Ciuti et al., 2014), our models did not detect a relationship between high densities of active oil and gas wells and alterations to the success of coyote predation on mule deer fawns, even at high predator density. Although we did not detect an effect of roads directly on recruitment in our models, it is likely that human modification of the landscape resulting from energy development shapes predator–prey relationships by influencing habitat quality and availability and through the introduction of novel landscape features such as roads and well pads (DeMars & Boutin, 2018; Guiden et al., 2019). In essence, given a year where proximate causes of mortality are high (e.g., high population density, low available habitat quality, and harsh seasonal weather) and the population is under duress, the contribution of high predator density and/or human disturbance appears to play a role in further limiting populations in that year but this does not drive the observed long‐term decline in fawn fall recruitment.

Documenting the relative importance of factors on population trends in ungulates can be a complex task with interactions among covariates (e.g., population density, habitat availability, climate, predators, and human disturbance) being difficult to disentangle (Bergman et al., 2015; Forrester & Wittmer, 2013; Gaillard et al., 2000; Peek, 1980; Sinclair & Krebs, 2002). Here we demonstrate the value of long‐term systematic wildlife monitoring datasets for understanding multivariate effects on wildlife population dynamics in a changing landscape. Quantifying and understanding the population‐level repercussions of disturbances and other contributing factors on animal life history, behavior, and physiology is a conservation priority across the world not unique to energy development. It is a requirement in many fields, such as game management, sustainable development, and as part of Environmental Impact Assessment (EIA), which is required by law in the European Union (European Habitats Directive 92/43/EEC) and United States (National Environmental Protection Act, 42 U.S.C. §§ 4321 *et seq*; Marine Mammal Protection Act, 16 U.S.C. §§ 1361 *et seq*.) legislation (King et al., 2015; Pirotta et al., 2018). Unfortunately, comprehensive risk assessments that encompass multivariate stressors on wildlife populations over time are rarely available due to a lack of long‐term data collection and lack of partnerships that facilitate the appropriate analysis of such data. This dataset identifies the long‐term relative importance and ranking of various factors that primarily drive this mule deer fawn population that is availability of quality habitat and severe weather, with human disturbance and predation playing lesser roles. Chronic wasting disease may soon become another primary driver of mule deer populations (Conner et al., 2021; Gillin & Mawdsley, 2018; Potapov et al., 2016).

Our paper utilizes mule deer fawn to female ratios (fawn:female) in fall as an index of fawns surviving the hazardous first 4–6 months of life where mortality causes such as abortion, malnutrition, drought, and predation must be confronted and survived. Attempts at predator control have largely been shown to be ineffective, costly and are increasingly being negatively viewed by the public (Ballard et al., 2001; Jensen et al., 2023). An important consideration here is the correlation between mule deer fawn fall recruitment (pre‐winter) and post‐winter survival relative to how it contributes to the adult population. Future research should focus on correlating overwinter fawn survival to fall fawn recruitment to allow wildlife managers to robustly use this index to predict future adult population recruitment. Rapidly changing ecological conditions and landscapes will inevitably make wildlife management even more difficult it would seem, therefore, that we need to follow a data‐driven approach to wildlife conservation and management, allowing robust analysis of long‐term monitoring data to explain the science in an empirical manner, and broaden our base of support for sound management (Prot, 2015). Management agencies and decision makers need to focus on aspects that can be controlled and have long‐term effects, such as maintaining quality habitat and mitigating human disturbance. As human populations continue to grow and develop supporting infrastructure (e.g., roads, housing and energy developments, and conversion of land from native vegetation to cropland) within and around wildlife habitat, we recognize the value of population monitoring schemes and the use of methodologies to understand the consequences of human development.

## AUTHOR CONTRIBUTIONS

**Kilian J Murphy:** Data curation (supporting); formal analysis (lead); investigation (lead); methodology (lead); project administration (lead); software (lead); validation (lead); visualization (equal); writing – original draft (lead); writing – review and editing (lead). **David R. Roberts:** Supervision (supporting); visualization (equal); writing – original draft (supporting); writing – review and editing (supporting). **William F Jensen:** Writing – original draft (supporting); writing – review and editing (supporting). **Scott E Nielsen:** Writing – original draft (supporting); writing – review and editing (supporting). **Sandra K. Johnson:** Data curation (equal). **Brian M. Hosek:** Data curation (equal). **Bruce Stillings:** Conceptualization (supporting); data curation (lead); writing – original draft (equal); writing – review and editing (equal). **Jesse L Kolar:** Data curation (equal); visualization (supporting); writing – original draft (equal); writing – review and editing (equal). **Mark Boyce:** Conceptualization (equal); data curation (supporting); writing – original draft (equal); writing – review and editing (equal). **Simone Ciuti:** Conceptualization (equal); data curation (lead); formal analysis (supporting); investigation (equal); methodology (supporting); project administration (supporting); supervision (lead); visualization (equal); writing – original draft (equal); writing – review and editing (equal).

## CONFLICT OF INTEREST STATEMENT

The authors have no conflict of interest to declare.

## FUNDING INFORMATION

Financial support for more than 50‐years of data collection, and for the analysis of these data, was provided by the North Dakota Game and Fish Department, Federal Aid Project W‐67‐R. No funding was acquired for the analysis and writing of the paper.

## Supporting information


Appendix S1

Figure S1

## Data Availability

All data used in the analysis for this project is saved in the open‐access repository Figshare, available here: https://figshare.com/articles/dataset/final_db_RDS/22298938, DOI:10.6084/m9.figshare.22298938

## References

[ece39976-bib-0001] Allen, M. C. , & Read, A. J. (2000). Habitat selection of foraging bottlenose dolphins in relation to boat density near Clearwater, Florida. Marine Mammal Science, 16, 815–824.

[ece39976-bib-0002] Allred, B. , Smith, W. , Twidwell, D. , Haggerty, J. , Running, S. , Naugle, D. , & Fuhlendorf, S. (2015). Ecosystem services lost to oil and gas in North America. Science, 348(6233), 401–402. 25908812 10.1126/science.aaa4785

[ece39976-bib-0003] Ballard, W. B. , Lutz, D. , Keegan, T. W. , Carpenter, L. H. , & deVos, J. C. (2001). Deer‐predator relationships: A review of recent north American studies with emphasis on mule and black‐tailed deer. Wildlife Society Bulletin, 29, 99–115.

[ece39976-bib-0004] Barton, K. (2022). MuMIn: multi‐model inference . R package version 1.47.1. 0. http://r‐forge.r‐project.org/projects/mumin/

[ece39976-bib-0005] Basille, M. , Fortin, D. , Dussault, C. , Ouellet, J. P. , & Courtois, R. (2013). Ecologically based definition of seasons clarifies predator–prey interactions. Ecography, 36(2), 220–229.

[ece39976-bib-0006] Baynard, C. , Mjachina, K. , Richardson, R. , Schupp, R. , Lambert, J. , & Chibilyev, A. (2017). Energy development in Colorado's Pawnee National Grasslands: Mapping and measuring the disturbance footprint of renewables and non‐renewables. Environmental Management, 59(6), 995–1016. 28374225 10.1007/s00267-017-0846-z

[ece39976-bib-0007] Benítez‐López, A. , Alkemade, R. , & Verweij, P. A. (2010). The impacts of roads and other infrastructure on mammal and bird populations: A meta‐analysis. Biological Conservation, 143(6), 1307–1316.

[ece39976-bib-0008] Berger, J. (2007). Fear, human shields and the redistribution of prey and predators in protected areas. Biology Letters, 3(6), 620–623. 17925272 10.1098/rsbl.2007.0415 PMC2391231

[ece39976-bib-0009] Bergman, E. J. , Doherty, P. F. , White, G. C. , & Holland, A. A. (2015). Density dependence in mule deer: A review of evidence. Wildlife Biology, 21(1), 18–29.

[ece39976-bib-0010] Bowyer, R. T. , Bleich, V. C. , Stewart, K. M. , Whiting, J. C. , & Monteith, K. L. (2014). Density dependence in ungulates: A review of causes and consequences, with some clarifications. California Fish and Game, 100, 550–572.

[ece39976-bib-0011] Bowyer, R. T. , Person, D. K. , & Pierce, B. M. (2005). Detecting top‐down versus bottom‐up regulation of ungulates by large carnivores: Implications for conservation of biodiversity. In J. C. Ray , K. H. Redford , R. S. Steneck , & J. Berger (Eds.), Large carnivores and the conservation of biodiversity (pp. 342–361). Island Press.

[ece39976-bib-0012] Bowyer, R. T. , Stewart, K. M. , Bleich, V. C. , Whiting, J. C. , Monteith, K. L. , Blum, M. E. , & LaSharr, T. N. (2020). Metrics of harvest for ungulate populations: Misconceptions, lurking variables, and prudent management. Alces, 56, 15–38.

[ece39976-bib-0013] Brittingham, M. C. , Maloney, K. O. , Farag, A. M. , Harper, D. D. , & Bowen, Z. H. (2014). Ecological risks of shale oil and gas development to wildlife, aquatic resources and their habitats. Environmental Science & Technology, 48(19), 11,034–11,047. 10.1021/es5020482 25188826

[ece39976-bib-0014] Brown, G. S. (2011). Patterns and causes of demographic variation in a harvested moose population: Evidence for the effects of climate and density‐dependent drivers. Journal of Animal Ecology, 80(6), 1288–1298. 21668892 10.1111/j.1365-2656.2011.01875.x

[ece39976-bib-0015] Burnham, K. P. , Anderson, D. R. , & Huyvaert, K. P. (2011). AIC model selection and multimodel inference in behavioral ecology: Some background, observations, and comparisons. Behavioral Ecology and Sociobiology, 65, 23–35.

[ece39976-bib-0016] Burr, P. C. (2014). Impacts of gas and oil development on sharp‐tailed grouse (Tympanuchus phasianellus) nest success and predator dynamics in western North Dakota . Master's Thesis, University of North Dakota, Grand Forks. https://commons.und.edu/theses/1625

[ece39976-bib-0017] Carrasco, M. , Barnosky, A. , & Graham, R. (2009). Quantifying the extent of north American mammal extinction relative to the pre‐anthropogenic baseline. PLoS One, 4(12), e8331 More than 75 percent decline over 27 years in total flying insect biomass in protected areas. 20016820 10.1371/journal.pone.0008331 PMC2789409

[ece39976-bib-0018] Christie, K. , Jensen, W. , Schmidt, J. , & Boyce, M. (2015). Long‐term changes in pronghorn abundance index linked to climate and oil development in North Dakota. Biological Conservation, 192, 445–453.

[ece39976-bib-0019] Ciuti, S. , Jensen, W. F. , Nielsen, S. E. , & Boyce, M. S. (2015). Predicting mule deer recruitment from climate oscillations for harvest management on the northern Great Plains. The Journal of Wildlife Management, 79, 1226–1238.

[ece39976-bib-0020] Ciuti, S. , Jensen, W. F. , Nielsen, S. E. , Boyce, M. S. , Johnson, S. K. , & Hosek, B. M. (2014). An evaluation of historical mule deer fawn recruitment in North Dakota. University of Alberta.

[ece39976-bib-0021] Ciuti, S. , Muhly, T. B. , Paton, D. G. , McDevitt, A. D. , Musiani, M. , & Boyce, M. S. (2012). Human selection of elk behavioural traits in a landscape of fear. Proceedings of the Royal Society B: Biological Sciences, 279(1746), 4407–4416. 10.1098/rspb.2012.1483 PMC3479801 22951744

[ece39976-bib-0022] Conner, M. M. , Wood, M. E. , Hubbs, A. , Binfet, J. , Holland, A. A. , Meduna, L. R. , Roug, A. , Runge, J. P. , Nordeen, T. D. , Pybus, M. J. , & Miller, M. W. (2021). The relationship between harvest management and chronic wasting disease prevalence trends in western mule deer (*Odocoileus hemionus*) herds. Journal of Wildlife Diseases., 57(4), 831–843. 34648639 10.7589/JWD-D-20-00226

[ece39976-bib-0023] Copeland, S. , Bradford, J. , Duniway, M. , & Schuster, R. (2017). Potential impacts of overlapping land‐use and climate in a sensitive dryland: A case study of the Colorado plateau, USA. The Bulletin of the Ecological Society of America, 98(4), 349–351.

[ece39976-bib-0024] Copping, A. , Battey, H. , Brown‐Saracino, J. , Massaua, M. , & Smith, C. (2014). An international assessment of the environmental effects of marine energy development. Ocean & Coastal Management, 99, 3–13.

[ece39976-bib-0025] Cressie, N. A. C. (1993). Statistics for spatial data. Wiley & Sons.

[ece39976-bib-0026] de la Torre, S. , Snowdon, C. T. , & Bejarano, M. (2000). Effects of human activities on wild pygmy marmosets in Ecuadorian Amazonia. Biological Conservation, 94, 153–163.

[ece39976-bib-0027] DeMars, C. A. , & Boutin, S. (2018). Nowhere to hide: Effects of linear features on predator–prey dynamics in a large mammal system. Journal of Animal Ecology, 87(1), 274–284. 28940254 10.1111/1365-2656.12760

[ece39976-bib-0028] Dickie, M. , McNay, S. R. , Sutherland, G. D. , Cody, M. , & Avgar, T. (2020). Corridors or risk? Movement along, and use of, linear features varies predictably among large mammal predator and prey species. Journal of Animal Ecology, 89(2), 623–634. 31648375 10.1111/1365-2656.13130 PMC7028095

[ece39976-bib-0029] Donnelly, S. , Cobbinah Wilson, I. , & Oduro Appiah, J. (2017). Comparing land change from shale gas infrastructure development in neighboring Utica and Marcellus regions, 2006–2015. Journal of Land Use Science, 12(5), 338–350.

[ece39976-bib-0030] Dormann, C. F. , Elith, J. , Bacher, S. , Buchmann, C. , Carl, G. , Carré, G. , Marquéz, J. R. G. , Gruber, B. , Lafourcade, B. , Leitão, P. J. , & Münkemüller, T. (2013). Collinearity: A review of methods to deal with it and a simulation study evaluating their performance. Ecography, 36(1), 27–46.

[ece39976-bib-0031] Doyle, D. , McNay, S. , Voller, J. , & Organizers . (1993). Deer status report. Western states and provinces deer workshop . Parksville, British Columbia, Canada. https://wafwa.org/wp‐content/uploads/2020/08/1993_WAFWA_DeerWorkshop_Proceedings.pdf

[ece39976-bib-0032] Dyer, S. J. , O'Neill, J. P. , Wasel, S. M. , & Boutin, S. (2001). Avoidance of industrial development by woodland caribou. Journal of Wildlife Management, 65, 531–542.

[ece39976-bib-0033] Ellrich, J. A. , Scrosati, R. A. , & Molis, M. (2015). Predator nonconsumptive effects on prey recruitment weaken with recruit density. Ecology, 96(3), 611–616. 26236858 10.1890/14-1856.1

[ece39976-bib-0034] Forrester, T. D. , & Wittmer, H. U. (2013). A review of the population dynamics of mule deer and black‐tailed deer *Odocoileus hemionus* in North America. Mammal Review, 43(4), 292–308.

[ece39976-bib-0035] Freddy, D. J. , Bronaugh, W. M. , & Fowler, M. C. (1986). Responses of mule deer to disturbance by persons afoot and snowmobiles. Wildlife Society Bulletin (1973–2006), 14(1), 63–68.

[ece39976-bib-0036] Frid, A. , & Dill, L. (2002). Human‐caused disturbance stimuli as a form of predation risk. Conservation Ecology, 6, 1.

[ece39976-bib-0037] Gaillard, J. M. , Festa‐Bianchet, M. , & Yoccoz, N. G. (1998). Population dynamics of large herbivores: Variable recruitment with constant adult survival. Trends in Ecology & Evolution, 13, 58–63. 21238201 10.1016/s0169-5347(97)01237-8

[ece39976-bib-0038] Gaillard, J. M. , Festa‐Bianchet, M. , Yoccoz, N. G. , Loison, A. , & Toigo, C. (2000). Temporal variation in fitness components and population dynamics of large herbivores. Annual Review of Ecology and Systematics, 31(1), 367–393.

[ece39976-bib-0039] Gamo, R. , & Beck, J. (2017). Energy disturbance and productivity of mule deer habitat in sage‐grouse Core areas. Rangeland Ecology & Management, 70(5), 576–583.

[ece39976-bib-0112] Gill, R. M. A. (1990). Monitoring of the status of European and North American cervid populations. United Nations Environment Programme (GEMS).

[ece39976-bib-0040] Gillin, C. M. , & Mawdsley, J. R. (Eds.). (2018). AFWA technical report on best management practices for surveillance, management and control of chronic wasting disease (p. 111). Association of Fish and Wildlife Agencies.

[ece39976-bib-0041] Graham, K. , Beckerman, A. , & Thirgood, S. (2005). Human–predator–prey conflicts: Ecological correlates, prey losses and patterns of management. Biological Conservation, 122(2), 159–171.

[ece39976-bib-0042] Guiden, P. , Bartel, S. , Byer, N. , Shipley, A. , & Orrock, J. (2019). Predator–prey interactions in the Anthropocene: Reconciling multiple aspects of novelty. Trends in Ecology & Evolution, 34(7), 616–627. 30902358 10.1016/j.tree.2019.02.017

[ece39976-bib-0113] Gullikson, B. S. (2019). Effects of Energy Development on Movements, Home Ranges, and Resource Selection of White‐Tailed Deer in the Western Dakotas. South Dakota State University.

[ece39976-bib-0043] Hallmann, C. , Sorg, M. , Jongejans, E. , Siepel, H. , Hofland, N. , Schwan, H. , Stenmans, W. , Müller, A. , Sumser, H. , Hörren, T. , Goulson, D. , & de Kroon, H. (2017). More than 75 percent decline over 27 years in total flying insect biomass in protected areas. PLoS One, 12(10), e0185809. 29045418 10.1371/journal.pone.0185809 PMC5646769

[ece39976-bib-0044] Hartig, M. F. (2021). Package ‘DHARMa’ R package CRAN .

[ece39976-bib-0045] Hixon, M. A. , & Webster, M. S. (2002). Density dependence in reef fish populations. In P. F. Sale (Ed.), Coral reef fishes: Dynamics and diversity in a complex ecosystem (pp. 303–325). Academic Press.

[ece39976-bib-0046] Hurley, M. A. , Unsworth, J. W. , Zager, P. , Hebblewhite, M. , Garton, E. O. , Montgomery, D. M. , Skalski, J. R. , & Maycock, C. L. (2011). Demographic response of mule deer to experimental reduction of coyotes and mountain lions in southeastern Idaho: Réponse Démographique du Cerf Mulet à la Réduction Expérimentale des Populations de Coyotes et de Pumas dans le Sud de l'Idaho. Wildlife Monographs, 178(1), 1–33.

[ece39976-bib-0047] Jensen, W. F. , Bleich, V. C. , & Whittaker, D. G. (2023). Historical trends in black‐tailed deer, mule deer, and their habitats. In Ecology and Management of Black‐tailed and Mule Deer of North America (pp. 25–42). CRC Press.

[ece39976-bib-0048] Jensen, W. F. (1988). Summer and fall ecology of mule deer in the North Dakota badlands . Dissertation, University of North Dakota, Grand Forks, USA.

[ece39976-bib-0049] Jensen, W. F. , Richardson, S. , & Stillings, B. (2022). Summary of incidental observations: Spring and fall mule deer surveys (1960‐2002) . P.R. report w‐67‐R‐62, C‐I. North Dakota Game and Fish Department, Bismarck, USA.

[ece39976-bib-0050] Karakoç, C. , Radchuk, V. , Harms, H. , & Chatzinotas, A. (2018). Interactions between predation and disturbances shape prey communities. Scientific Reports, 8, 1. 29445181 10.1038/s41598-018-21219-x PMC5813231

[ece39976-bib-0051] King, S. L. , Schick, R. S. , Donovan, C. , Booth, C. G. , Burgman, M. , Thomas, L. , & Harwood, J. (2015). An interim framework for assessing the population consequences of disturbance. Methods in Ecology and Evolution, 6(10), 1150–1158.

[ece39976-bib-0052] Knue, J. (1991). Big game in North Dakota: A short history. North Dakota State Game & Fish.

[ece39976-bib-0053] Kolar, J. L. , Millspaugh, J. J. , Stillings, B. A. , Hansen, C. P. , Chitwood, D. , Rota, D. T. , & Skelly, B. P. (2017). Potential effects of oil and gas energy development on mule deer in western North Dakota . Study No. C‐X: Report No. A‐258. North Dakota game and fish department, Bismarck, USA.

[ece39976-bib-0054] Laundré, J. W. , Hernández, L. , & Ripple, W. J. (2010). The landscape of fear: Ecological implications of being afraid. The Open Ecology Journal, 3, 1–7.

[ece39976-bib-0055] Laycoek, W. A. , & Price, D. A. (1970). Factors influencing forage quality: Environmental influences on nutritional value of forage plants . Range and wildlife habitat evaluation, a research symposium. U.S. dep. Agr. Forest Serv. Misc. pub. 1147, 3747.

[ece39976-bib-0056] Lingle, S. , Feldman, A. , Boyce, M. S. , & Wilson, W. F. (2008). Prey behavior, age‐dependent vulnerability, and predation rates. The American Naturalist, 172(5), 712–725. 10.1086/591675 18840071

[ece39976-bib-0057] Loehle, C. , & Eschenbach, W. (2011). Historical bird and terrestrial mammal extinction rates and causes. Diversity and Distributions, 18(1), 84–91.

[ece39976-bib-0058] Lutz, D. W. , Heffelfinger, J. R. , Tessmann, S. A. , Gamo, R. S. , & Siegel, S. (2011). Energy development guidelines for mule deer. Western Association of Fish and Wildlife Agencies.

[ece39976-bib-0059] Lynch, E. , Northrup, J. M. , McKenna, M. F. , Anderson, C. R., Jr. , Angeloni, L. , & Wittemyer, G. (2015). Landscape and anthropogenic features influence the use of auditory vigilance by mule deer. Behavioural Ecology, 26(1), 75–82.

[ece39976-bib-0060] Muhly, T. B. , Semeniuk, C. , Massolo, A. , Hickman, L. , & Musiani, M. (2011). Human activity helps prey win the predator‐prey space race. PLoS One, 6(3), e17050. 21399682 10.1371/journal.pone.0017050 PMC3047538

[ece39976-bib-0061] Murphy, A. , Diefenbach, D. R. , Ternent, M. , Lovallo, M. , & Miller, D. (2021). Threading the needle: How humans influence predator–prey spatiotemporal interactions in a multiple‐predator system. Journal of Animal Ecology, 90(10), 2377–2390. 34048031 10.1111/1365-2656.13548

[ece39976-bib-0062] Murphy, K. J. , Morera‐Pujol, V. , Ryan, E. , Byrne, A. W. , Breslin, P. , & Ciuti, S. (2022). Habitat availability alters the relative risk of a bovine tuberculosis breakdown in the aftermath of a commercial forest clearfell disturbance. Journal of Applied Ecology, 59(9), 2333–2345.

[ece39976-bib-0063] Naugle, D. E. , Doherty, K. E. , Walker, B. L. , Holloran, M. J. , & Copeland, H. E. (2011). Energy development and greater sage‐grouse. In S. T. Knick & J. W. Connelly (Eds.), Greater sage‐grouse: Ecology and conservation of a landscape species and its habitats. Stud. Avian biol. Vol 38 (pp. 489–502). University of California Press.

[ece39976-bib-0064] Nicholson, M. C. , Bowyer, R. T. , & Kie, J. G. (2006). Forage selection by mule deer: Does niche breadth increase with population density? Journal of Zoology, 269(1), 39–49.

[ece39976-bib-0065] North Dakota Department of Mineral Resources (NDDMR) . (2021). GIS map server. North Dakota oil and gas division . https://www.dmr.nd.gov/OaGIMS/viewer.htm

[ece39976-bib-0066] Northrup, J. M. , & Wittemyer, G. (2013). Characterising the impacts of emerging energy development on wildlife, with an eye towards mitigation. Ecology Letters, 16(1), 112–125. 23013218 10.1111/ele.12009

[ece39976-bib-0068] Parker, K. L. , Gillingham, M. P. , Hanley, T. A. , & Robbins, C. T. (1996). Foraging efficiency: Energy expenditure versus energy gain in free‐ranging black‐tailed deer. Canadian Journal of Zoology, 74, 442–450.

[ece39976-bib-0069] Peek, J. M. (1980). Natural regulation of ungulates. Wildlife Society Bulletin, 8, 217–227.

[ece39976-bib-0070] Peek, J. M. , Dennis, B. , & Hershey, T. (2002). Predicting population trends of mule deer. Journal of Wildlife Management, 66, 729–736.

[ece39976-bib-0071] Pirotta, E. , Booth, C. G. , Costa, D. P. , Fleishman, E. , Kraus, S. D. , Lusseau, D. , Moretti, D. , New, L. F. , Schick, R. S. , Schwarz, L. K. , & Simmons, S. E. (2018). Understanding the population consequences of disturbance. Ecology and Evolution, 8(19), 9934–9946. 30386587 10.1002/ece3.4458 PMC6202709

[ece39976-bib-0072] Potapov, A. , Merrill, E. , Pybus, M. , & Lewis, M. A. (2016). Chronic wasting disease: Transmission mechanisms and the possibility of harvest management. PLoS One, 10(10), e0140024. 10.1371/journal.pone.0151039 PMC4786122 26963921

[ece39976-bib-0073] Preisser, E. L. , & Bolnick, D. I. (2008). The many faces of fear: Comparing the pathways and impacts of nonconsumptive predator effects on prey populations. PLoS One, 3(6), e2465. 18560575 10.1371/journal.pone.0002465 PMC2409076

[ece39976-bib-0074] Preston, T. M. , & Kim, K. (2016). Land cover changes associated with recent energy development in the Williston Basin; Northern Great Plains, USA. Science of the Total Environment, 566, 1511–1518. 27318516 10.1016/j.scitotenv.2016.06.038

[ece39976-bib-0075] Prot, S. (2015). Science denial as intergroup conflict: Using social identity theory, intergroup emotions theory and intergroup threat theory to explain angry denial of science . Dissertation, Iowa State University, Ames, USA.

[ece39976-bib-0076] R Development Core Team . (2021). R: A language and environment for statistical computing. R Foundation for Statistical Computing.

[ece39976-bib-0077] Revelle, W. (2021). Psych: Procedures for psychological, psychometric, and personality research . Northwestern University, Evanston, Illinois, R package version 2.1.9. https://CRAN.R‐project.org/package=psych

[ece39976-bib-0078] Sæther, B. E. (1997). Environmental stochasticity and population dynamics of large herbivores: A search for mechanisms. Trends in Ecology & Evolution, 12(4), 143–149. 21238011 10.1016/s0169-5347(96)10068-9

[ece39976-bib-0079] Sawyer, H. , Kauffman, M. J. , & Nielson, R. M. (2009). Influence of well pad activity on winter habitat selection patterns of mule deer. Journal of Wildlife Management, 73, 1052–1061.

[ece39976-bib-0080] Sawyer, H. , Korfanta, N. , Nielson, R. , Monteith, K. , & Strickland, D. (2017). Mule deer and energy development‐long‐term trends of habituation and abundance. Global Change Biology, 23(11), 4521–4529. 28375581 10.1111/gcb.13711

[ece39976-bib-0116] Sawyer, H. , & Lindzey, F. (2004). Assessing impacts of oil and gas development on mule deer. Transactions of the Sixty‐Ninth North American Wildlife and Natural Resources Conference, 69, 267–278.

[ece39976-bib-0081] Sawyer, H. , Lindzey, F. , McWhirter, D. , & Andrews, K. (2002). Potential effects of oil and gas development on mule deer and pronghorn populations in Western Wyoming. Transactions of the Sixty‐Seventh North American Wildlife and Natural Resource Conference, 67, 350–365.

[ece39976-bib-0082] Sawyer, H. , Nielson, R. M. , Lindzey, F. , & McDonald, L. L. (2006). Winter habitat selection of mule deer before and during development of a natural gas field. Journal of Wildlife Management, 70, 396–403.

[ece39976-bib-0083] Sikes, R. S. , & Animal Care and Use Committee of the American Society of Mammalogists . (2016). 2016 Guidelines of the American Society of Mammalogists for the use of wild mammals in research and education. Journal of mammalogy, 97(3), 663–688. 29692469 10.1093/jmammal/gyw078 PMC5909806

[ece39976-bib-0085] Sinclair, A. R. E. , & Krebs, C. J. (2002). Complex numerical responses to top–down and bottom–up processes in vertebrate populations. Philosophical Transactions of the Royal Society of London. Series B: Biological Sciences, 357(1425), 1221–1231. 12396514 10.1098/rstb.2002.1123 PMC1693037

[ece39976-bib-0086] Souther, S. , Tingley, M. , Popescu, V. , Hayman, D. , Ryan, M. , Graves, T. , Hartl, B. , & Terrell, K. (2014). Biotic impacts of energy development from shale: Research priorities and knowledge gaps. Frontiers in Ecology and the Environment, 12(6), 330–338.

[ece39976-bib-0087] Stewart, K. M. , Bowyer, R. T. , Dick, B. L. , & Kie, J. G. (2011). Effects of density dependence on diet composition of north American elk Cervus elaphus and mule deer *Odocoileus hemionus*: An experimental manipulation. Wildlife Biology, 17(4), 417–430.

[ece39976-bib-0088] Tanner, J. T. (1966). Effects of population density on growth rates of animal populations. Ecology, 47(5), 733–745.

[ece39976-bib-0089] Torbit, S. C. , Carpenter, L. H. , Alldredge, A. W. , & Swift, D. M. (1985). Mule deer body‐composition—A comparison of methods. Journal of Wildlife Management, 49, 86–91.

[ece39976-bib-0090] Torbit, S. C. , Carpenter, L. H. , Swift, D. M. , & Alldredge, A. W. (1985). Differential loss of fat and protein by mule deer during winter. Journal of Wildlife Management, 49, 80–85.

[ece39976-bib-0091] Trainor, A. , McDonald, R. , & Fargione, J. (2016). Energy sprawl is the largest driver of land use change in the United States. PLoS One, 11(9), e0162269. 27607423 10.1371/journal.pone.0162269 PMC5015902

[ece39976-bib-0092] Venables, W. N. , & Ripley, B. D. (2002). Modern applied statistics with S (4th ed.). Springer‐Verlag.

[ece39976-bib-0093] Vye, S. , Emmerson, M. , Dick, J. , & O'Connor, N. (2017). Cumulative effects of multiple stressors: An invasive oyster and nutrient enrichment reduce subsequent invasive barnacle recruitment. Journal of Experimental Marine Biology and Ecology, 486, 322–327.

[ece39976-bib-0094] Walker, B. , Neubaum, M. , Goforth, S. , & Flenner, M. (2019). Quantifying habitat loss and modification from recent expansion of energy infrastructure in an isolated, peripheral greater sage‐grouse population. Journal of Environmental Management, 255, 109819. 31756579 10.1016/j.jenvman.2019.109819

[ece39976-bib-0095] Wallmo, O. C. (1981). Mule and black‐tailed deer of North America USA. University of Nebraska Press.

[ece39976-bib-0096] Walther, F. (1969). Flight behaviour and avoidance of predators in Thomson's gazelle (Gazella Thomsoni Guenther 1884). Behaviour, 34(3), 184–220.

[ece39976-bib-0097] Whittaker, D. G. , & Lindzey, F. G. (1999). Effect of coyote predation on early fawn survival in sympatric deer species. Wildlife Society Bulletin, 27(2), 256–262.

[ece39976-bib-0098] Wood, S. N. (2006). On confidence intervals for generalized additive models based on penalized regression splines. Australian & New Zealand Journal of Statistics, 48(4), 445–464.

[ece39976-bib-0099] Wood, S. N. (2017). Generalized additive models: An introduction with R (2nd ed.). Chapman and Hall/CRC.

[ece39976-bib-0119] Woodroffe, R. , Thirgood, S. , & Rabinowitz, A. (eds.). (2005). People and wildlife, conflict or co‐existence? (Vol. 9). Cambridge University Press.

[ece39976-bib-0100] Ziegler, D. , & Myers, W. (1983). Proceedings of the mule deer workshop. Washington Department of Game https://wafwa.org/wp‐content/uploads/2020/08/1983_WAFWA_DeerWorkshop_Proceedings.pdf.

[ece39976-bib-0101] Zuur, A. F. , Ieno, E. N. , Walker, N. J. , Saveliev, A. A. , & Smith, G. M. (2009). Mixed effects models and extensions in ecology with R Vol. 574. Springer.

