# Supplementary material for: Mule deer fawn recruitment dynamics in an energy disturbed landscape
Source: Ecol Evol. 2023 Apr 19;13(4):e9976. doi: 10.1002/ece3.9976 (PMC10116077; doi:10.1002/ece3.9976)
Supplement: Supplementary file 1 — Appendix S1 [file ECE3-13-e9976-s001.pdf]

SM1

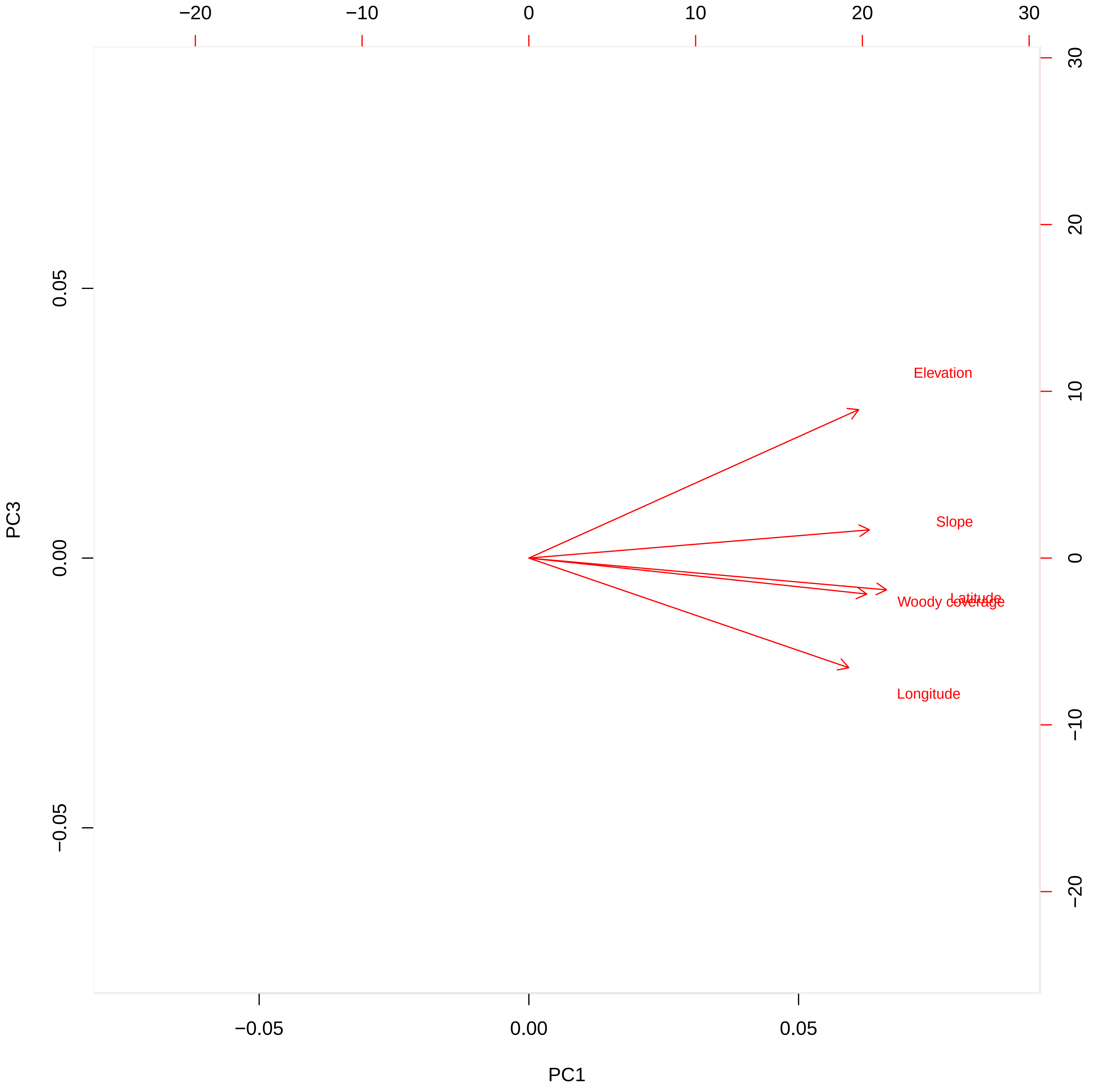

−20
−10
0
10
20
30
30
20
10
0
−10
−20
0.05
0.00
−0.05
PC3
Elevation
Slope
Latitude
Woody coverage
Longitude
−0.05
0.00
0.05
PC1

SM2

A

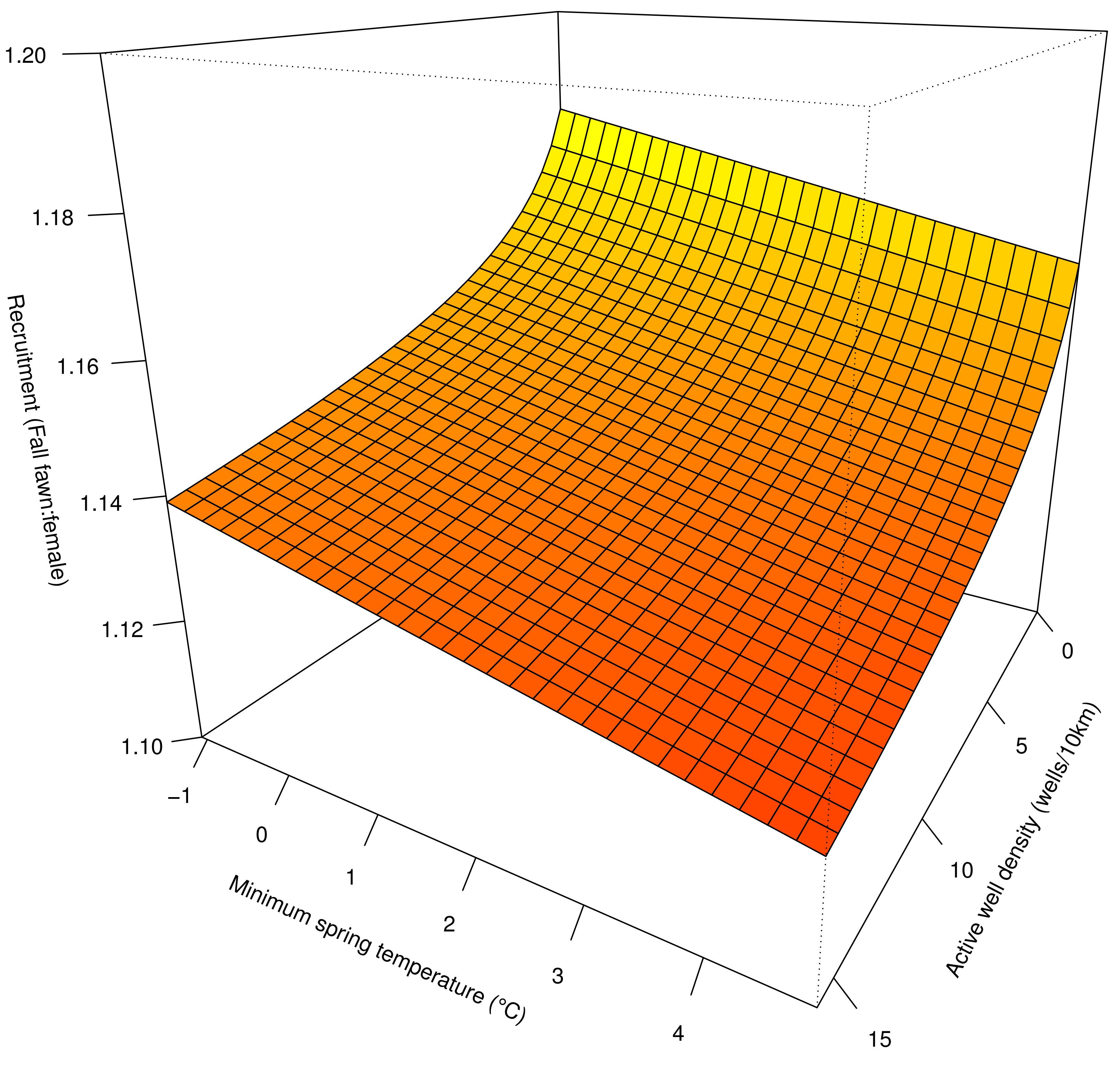

1.20
1.18
1.16
1.14
1.12
1.10
Recruitment (Fall fawn:female)
−1
0
1
2
3
4
Minimum spring temperature (°C)
0
5
10
15
Active well density (wells/10km)

SM2

B

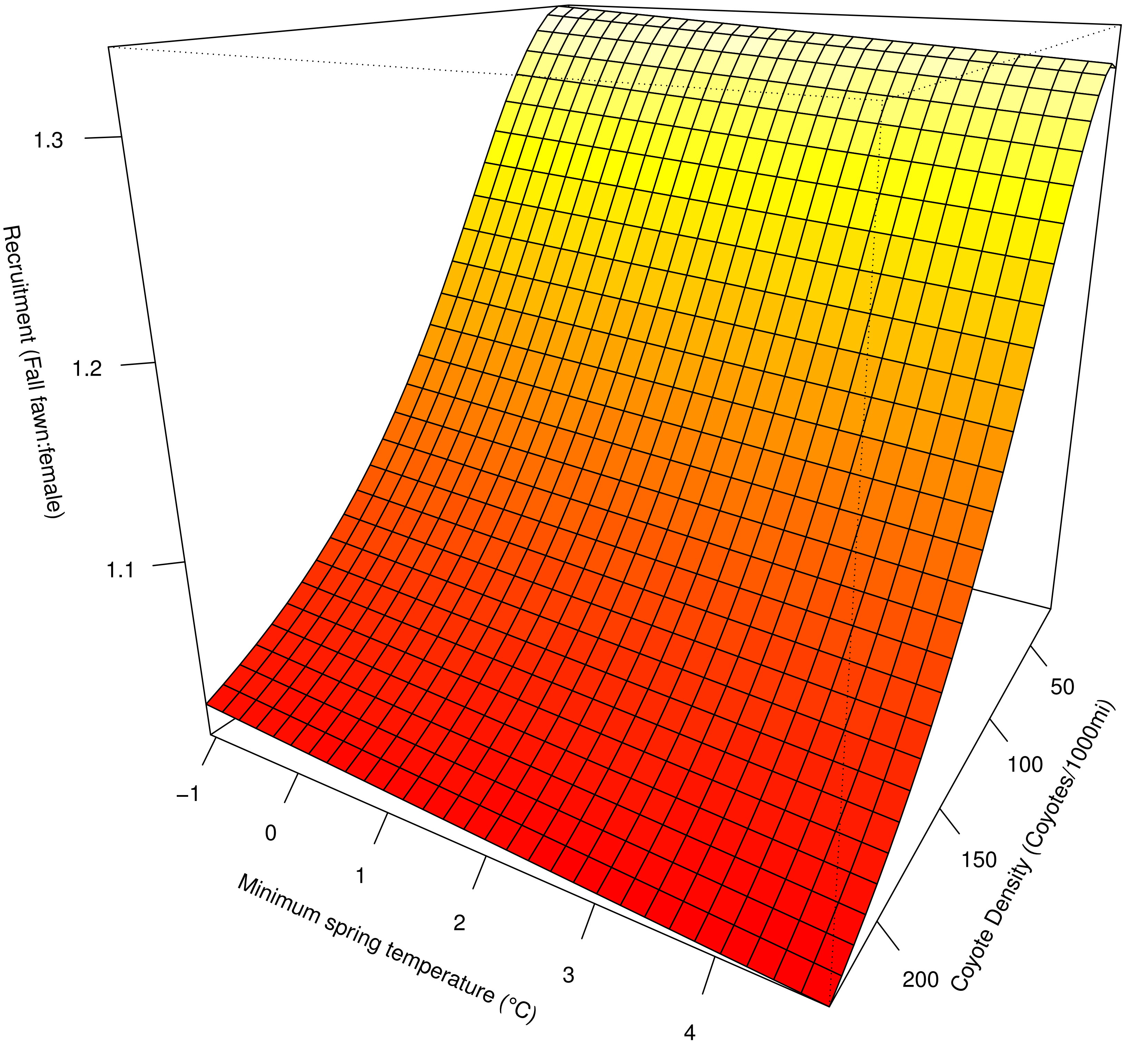
1.3
1.2
1.1
Recruitment (Fall fawn:female)
−1
0
1
2
3
4
Minimum spring temperature (°C)
50
100
150
200
Coyote Density (Coyotes/1000mi)

SM3

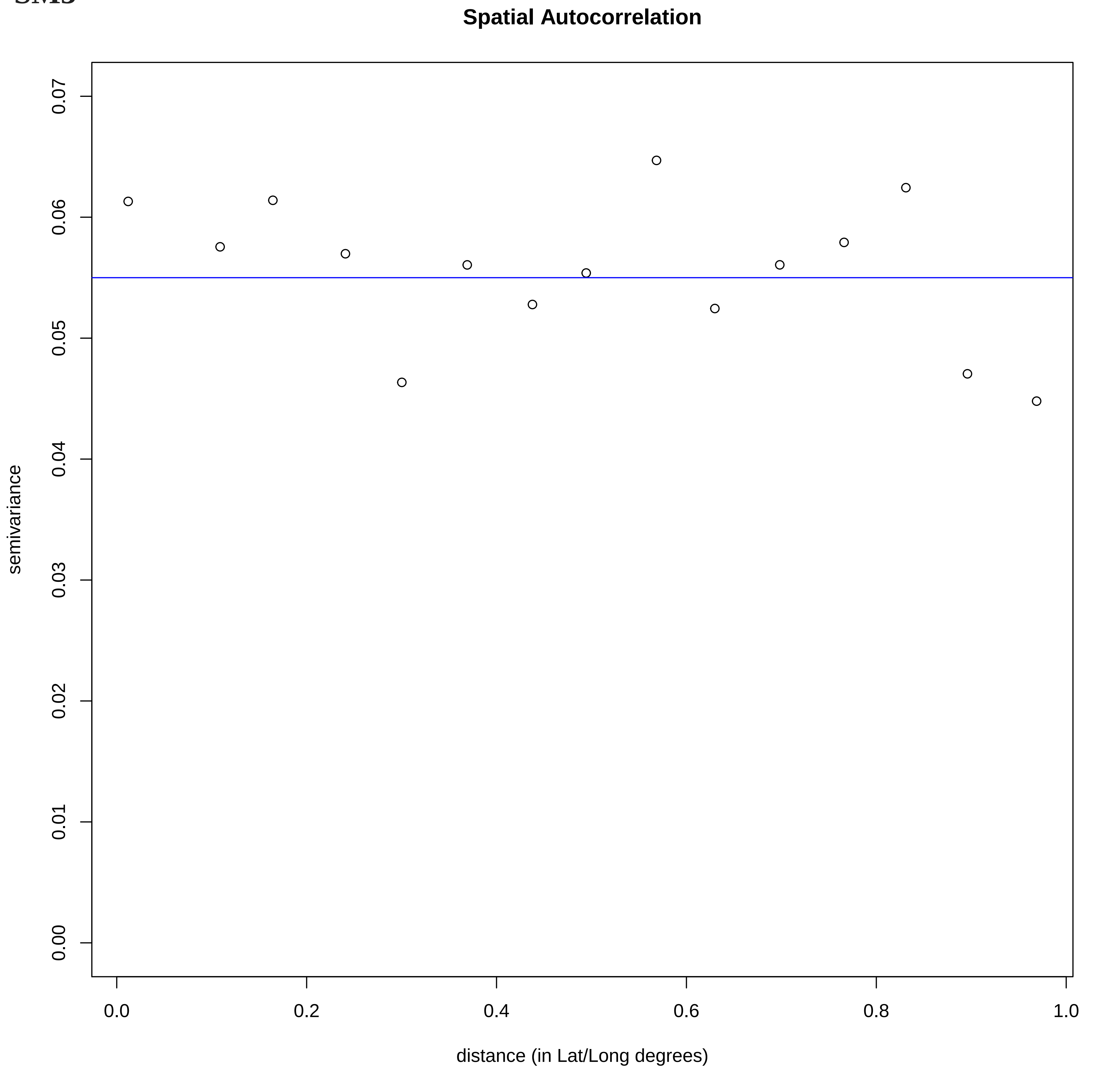

Spatial Autocorrelation
semivariance
distance (in Lat/Long degrees)
0.00
0.01
0.02
0.03
0.04
0.05
0.06
0.07
0.0
0.2
0.4
0.6
0.8
1.0

SM4

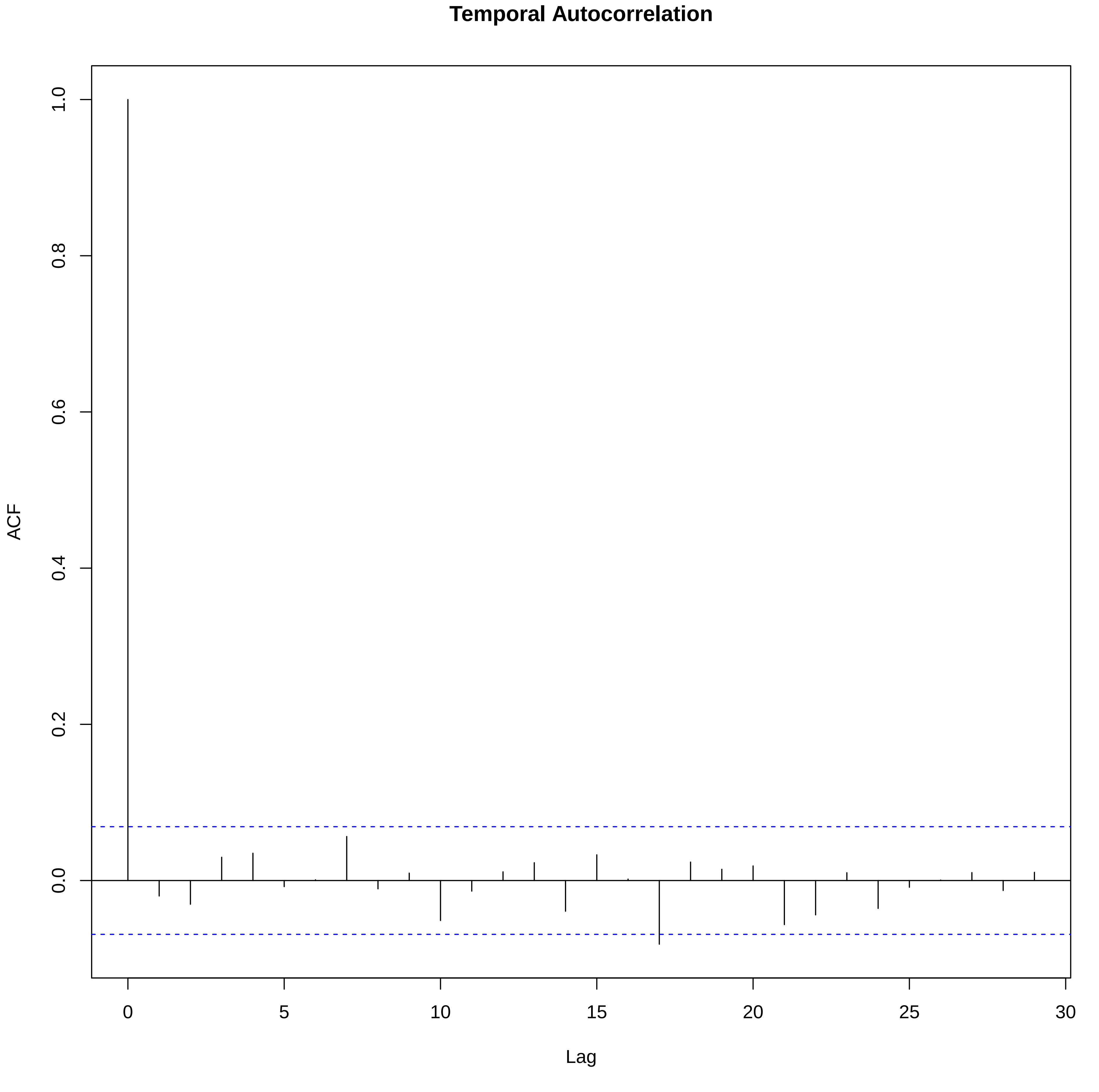
Temporal Autocorrelation
ACF
Lag
0.0
0.2
0.4
0.6
0.8
1.0
0
5
10
15
20
25
30

SM5

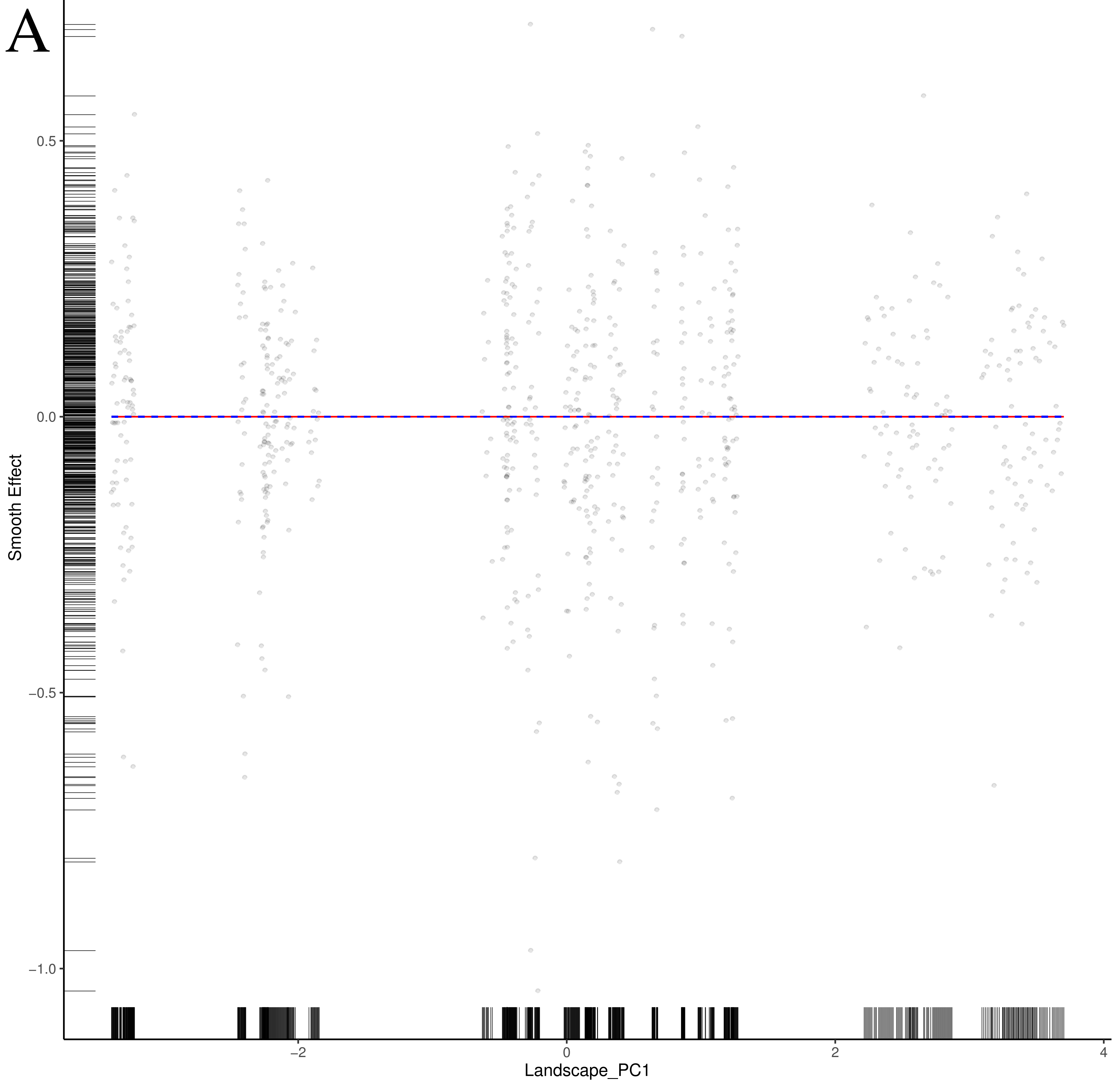

SM5

B

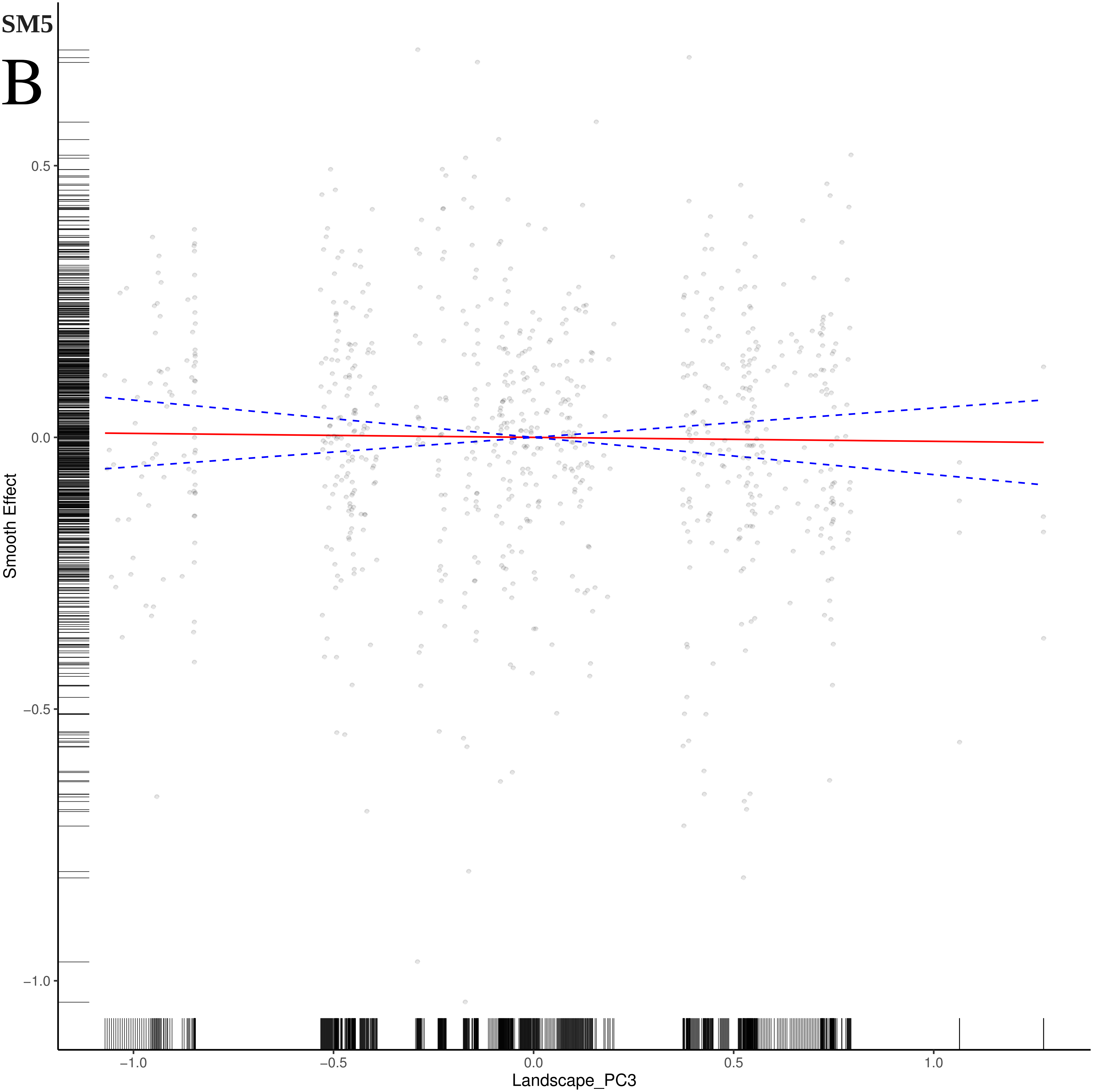

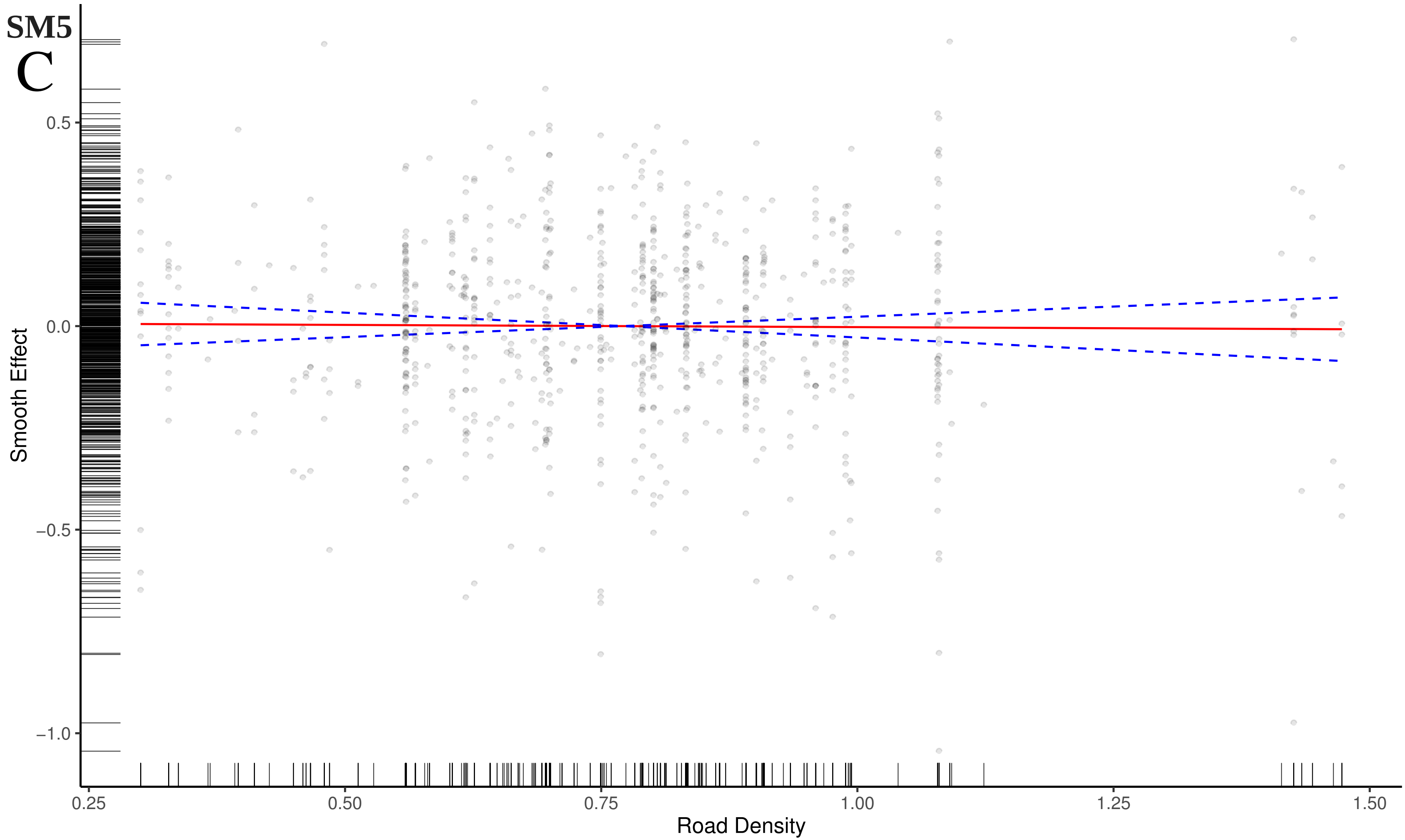
SM5
C
0.5
0.0
−0.5
−1.0
Smooth Effect
0.25
0.50
0.75
1.00
1.25
1.50
Road Density

SM5

D

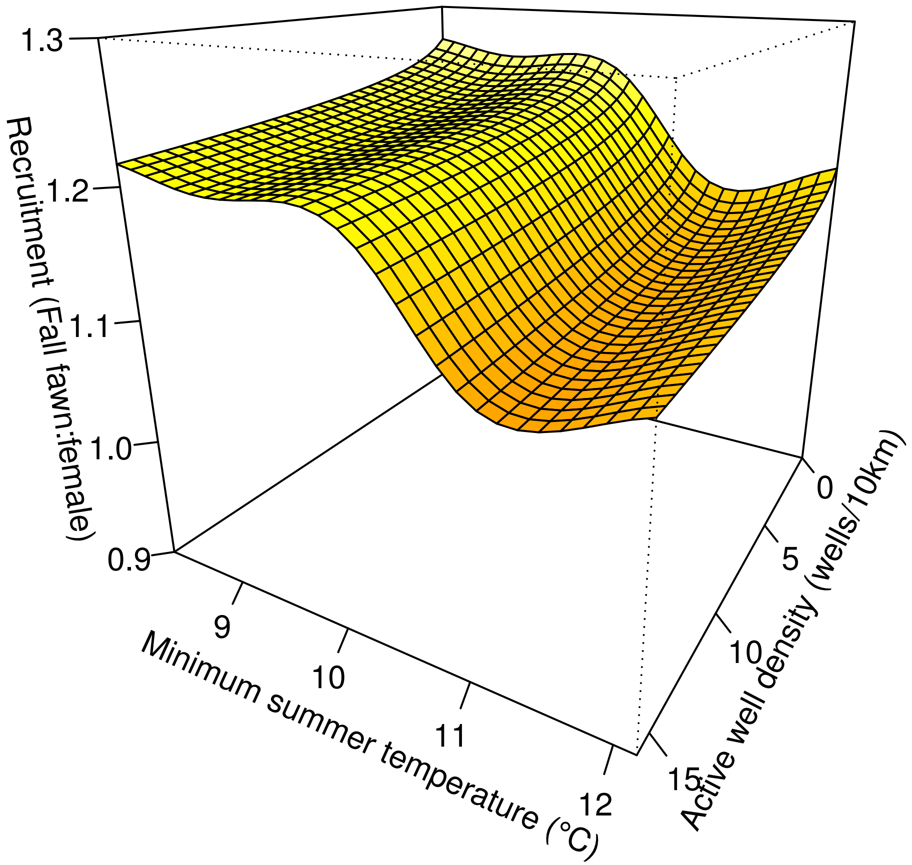

1.3
1.2
1.1
1.0
0.9
Recruitment (Fall fawn:female)
9
10
11
12
Minimum summer temperature (°C)
0
5
10
15
Active well density (wells/10km)

SM5

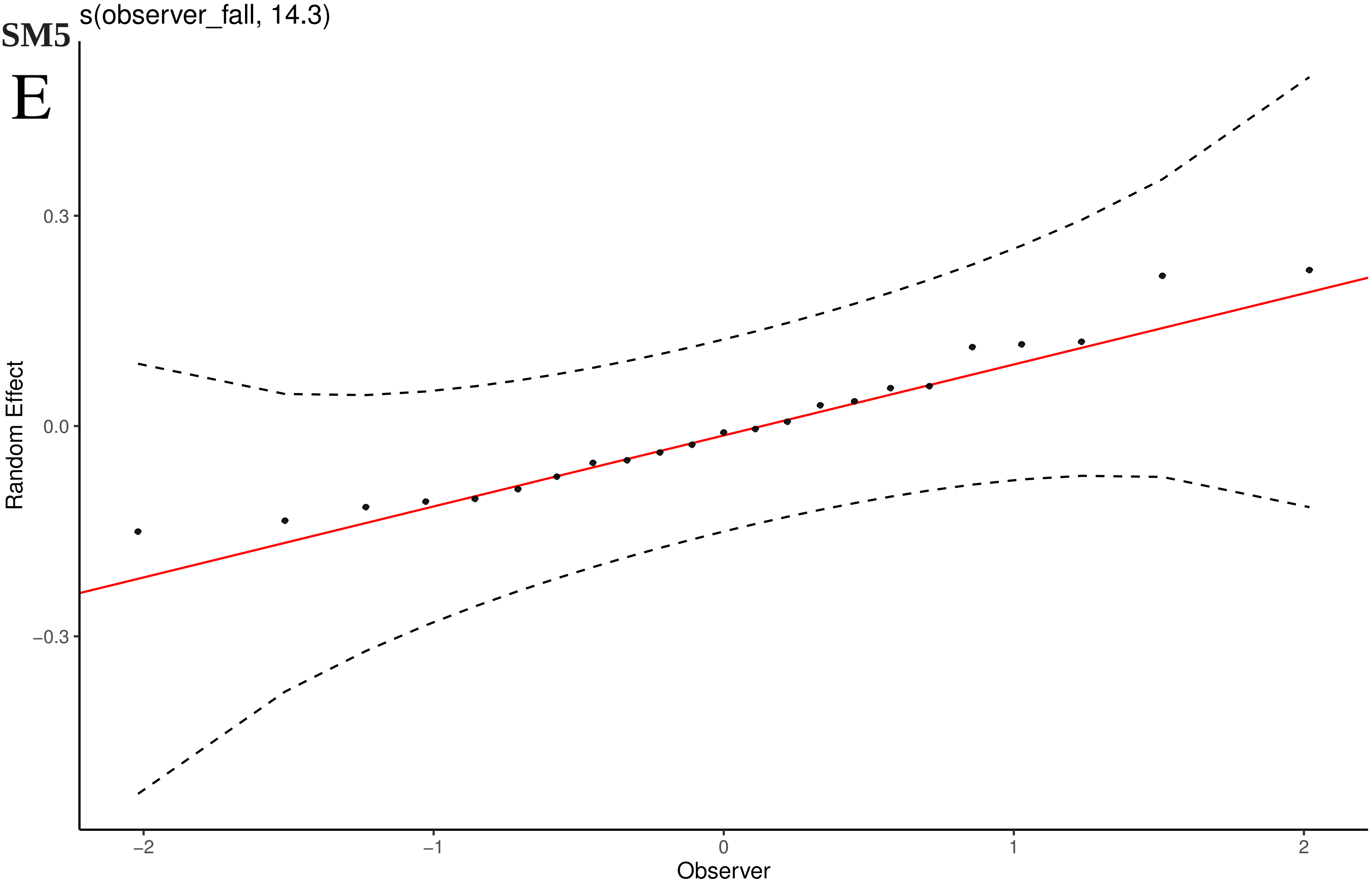

SM5

F

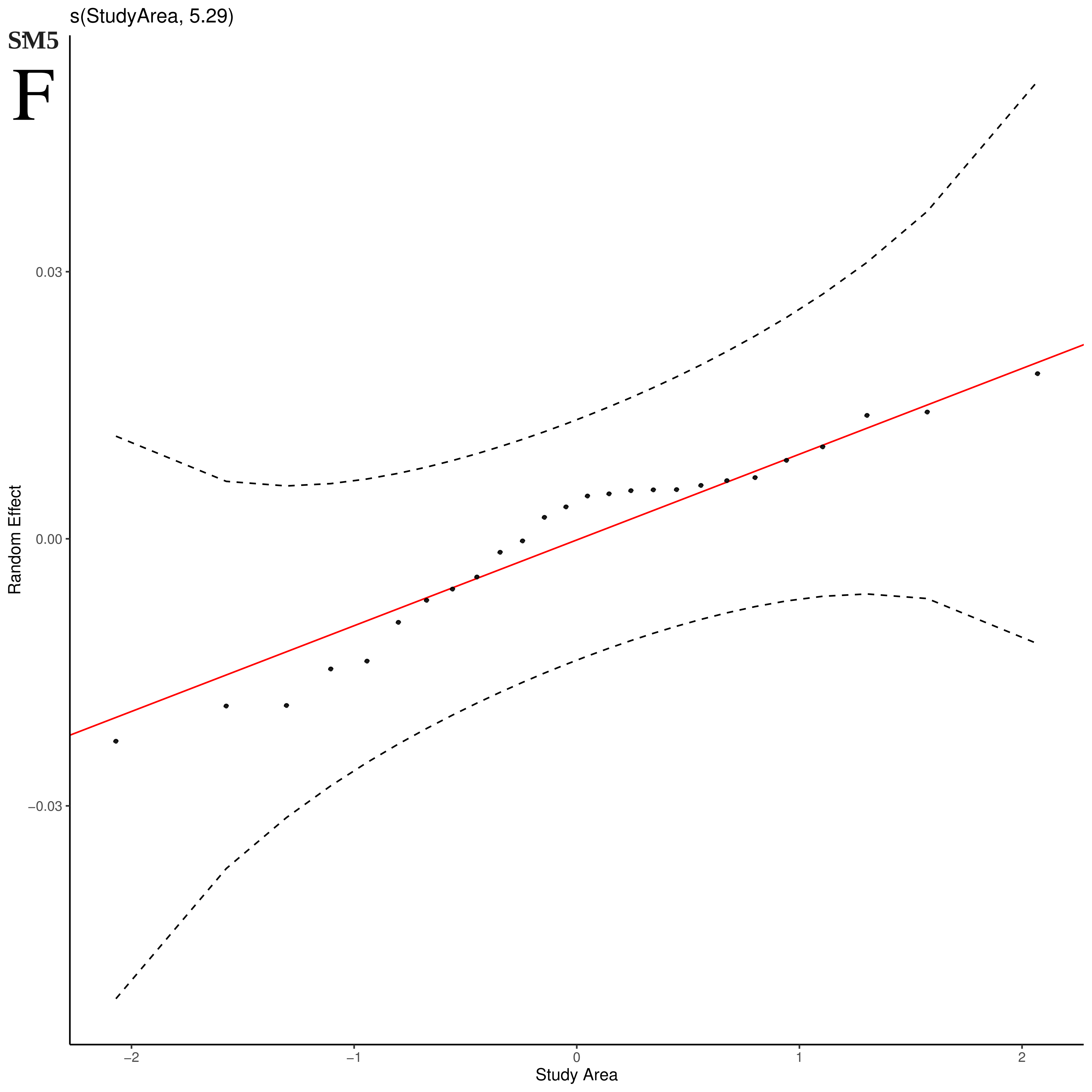

SM1: Biplot for principle component analysis of landscape predictors included in our models. PC1 represents study sites in the north-east that have experienced encroachment of Rocky Mountain juniper whereas PC3 (south-west) accounts for study sites that have rugged terrain and less encroachment of woody vegetation.

SM2: Interaction plots for the second ranked generalised additive mixed effect model explaining fawn recruitment in North Dakota from 1962 to 2012, which included average minimum temperature in spring instead of average spring snow depth as our spring weather predictor. Note: only predictors that are different from the top ranked model are included here: (A) Interaction between average minimum spring temperature and active well density; (B) Interaction between average minimum spring temperature and coyote density

SM3: Auto-correlation function plot of the residuals for the top ranked generalised additive mixed effect model explaining fawn recruitment in North Dakota from 1962 to 2012.

SM4: Variogram for the top ranked generalised additive mixed effect model explaining fawn recruitment in North Dakota from 1962 to 2012.

SM5: Smooth term plots for the top ranked generalised additive mixed effect model explaining fawn recruitment in North Dakota from 1962 to 2012. Note: only predictors not shown in the main manuscript are included here: landscape PC1 (A), landscape PC3 (B), road density ©, interaction of well density and average minimum summer temperature (D), observer random effect (E), study area random effect (F).
