# Supplementary material for: Mule deer fawn recruitment dynamics in an energy disturbed landscape
Source: Ecol Evol. 2023 Apr 19;13(4):e9976. doi: 10.1002/ece3.9976 (PMC10116077; doi:10.1002/ece3.9976)
Supplement: Supplementary file 2 — Figure S1 [file ECE3-13-e9976-s002.pdf]

A

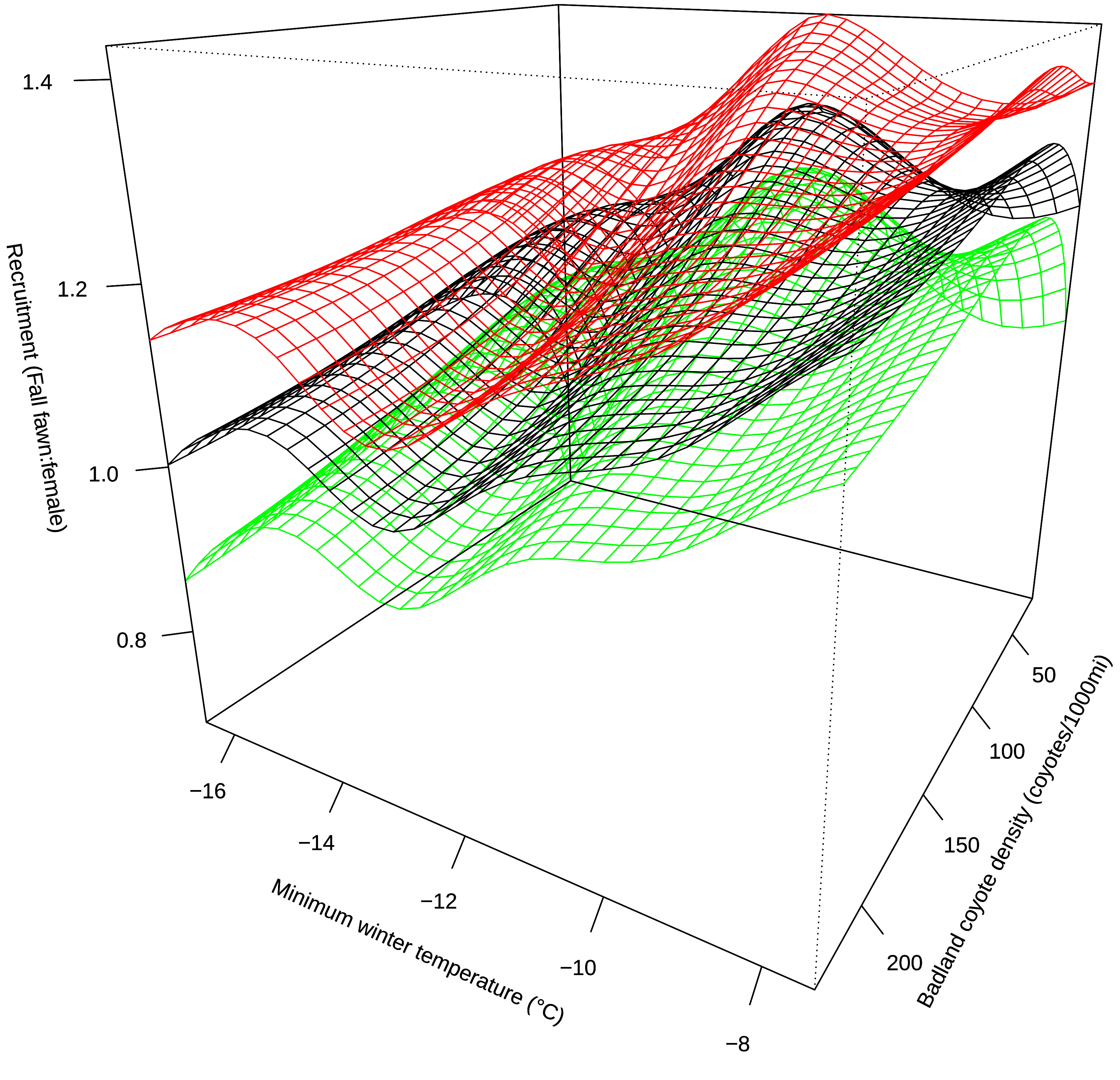

red/green are +/− 1.96 s.e.

B

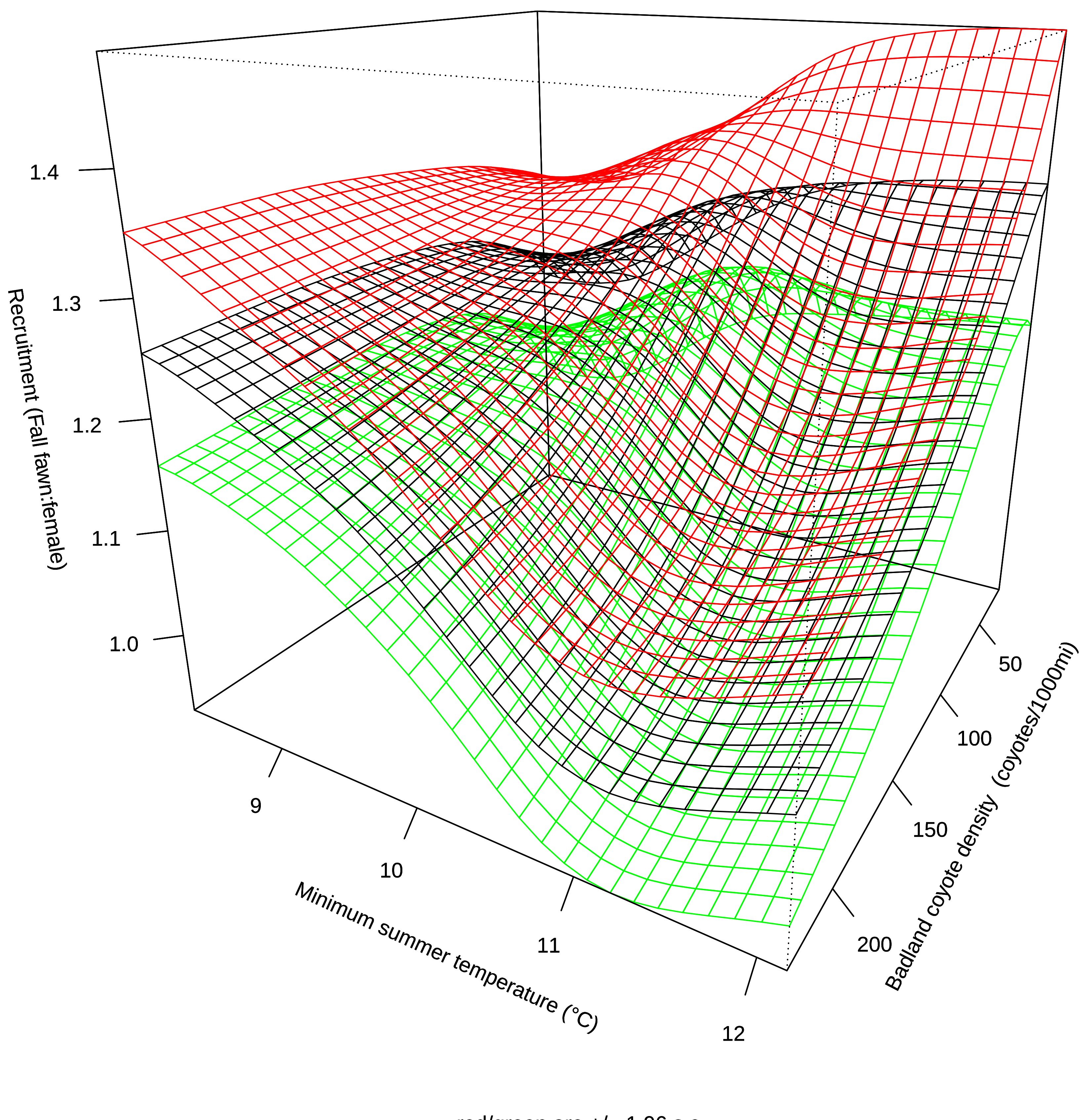

red/green are +/− 1.96 s.e.

C

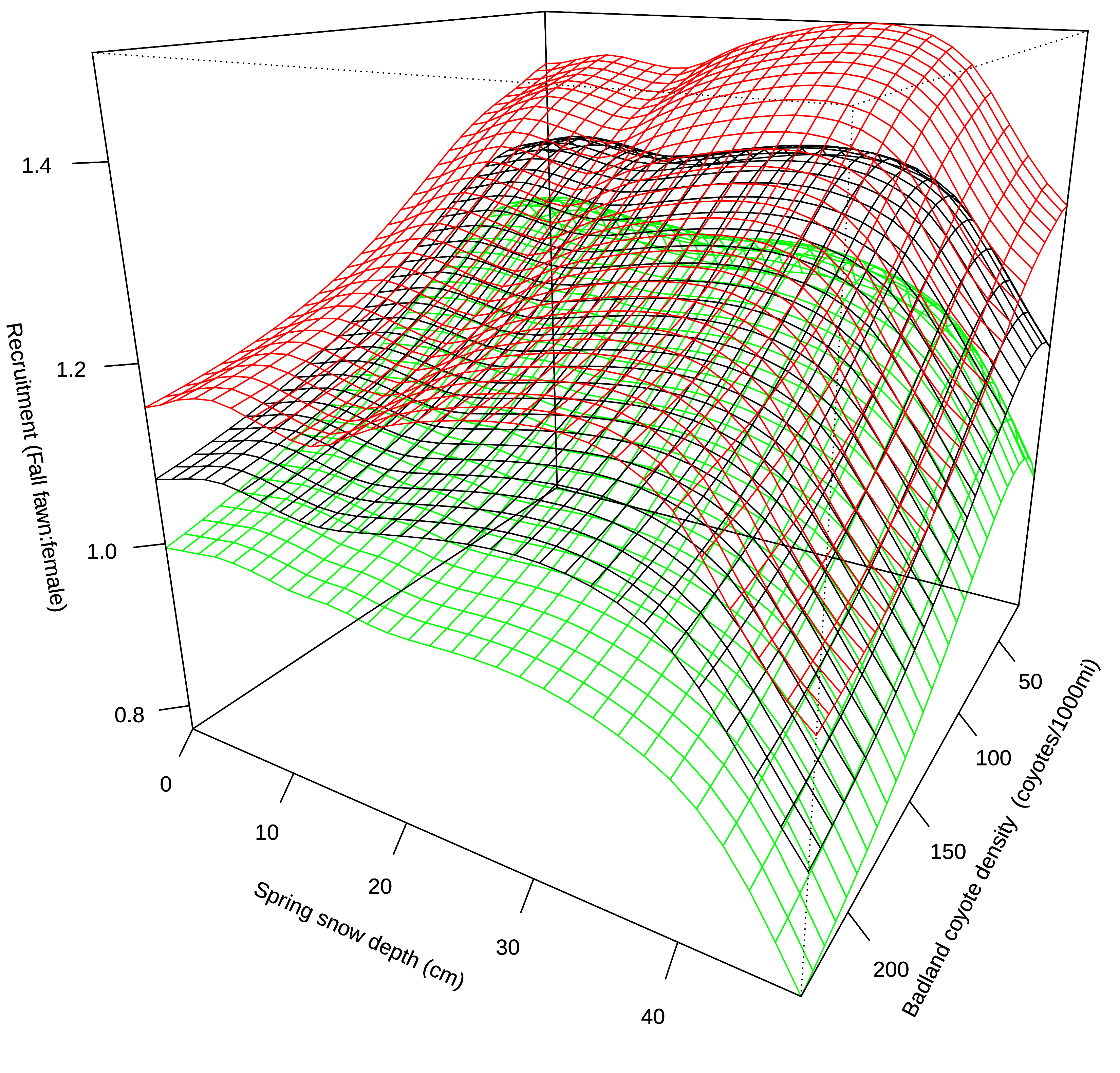

red/green are +/− 1.96 s.e.

D

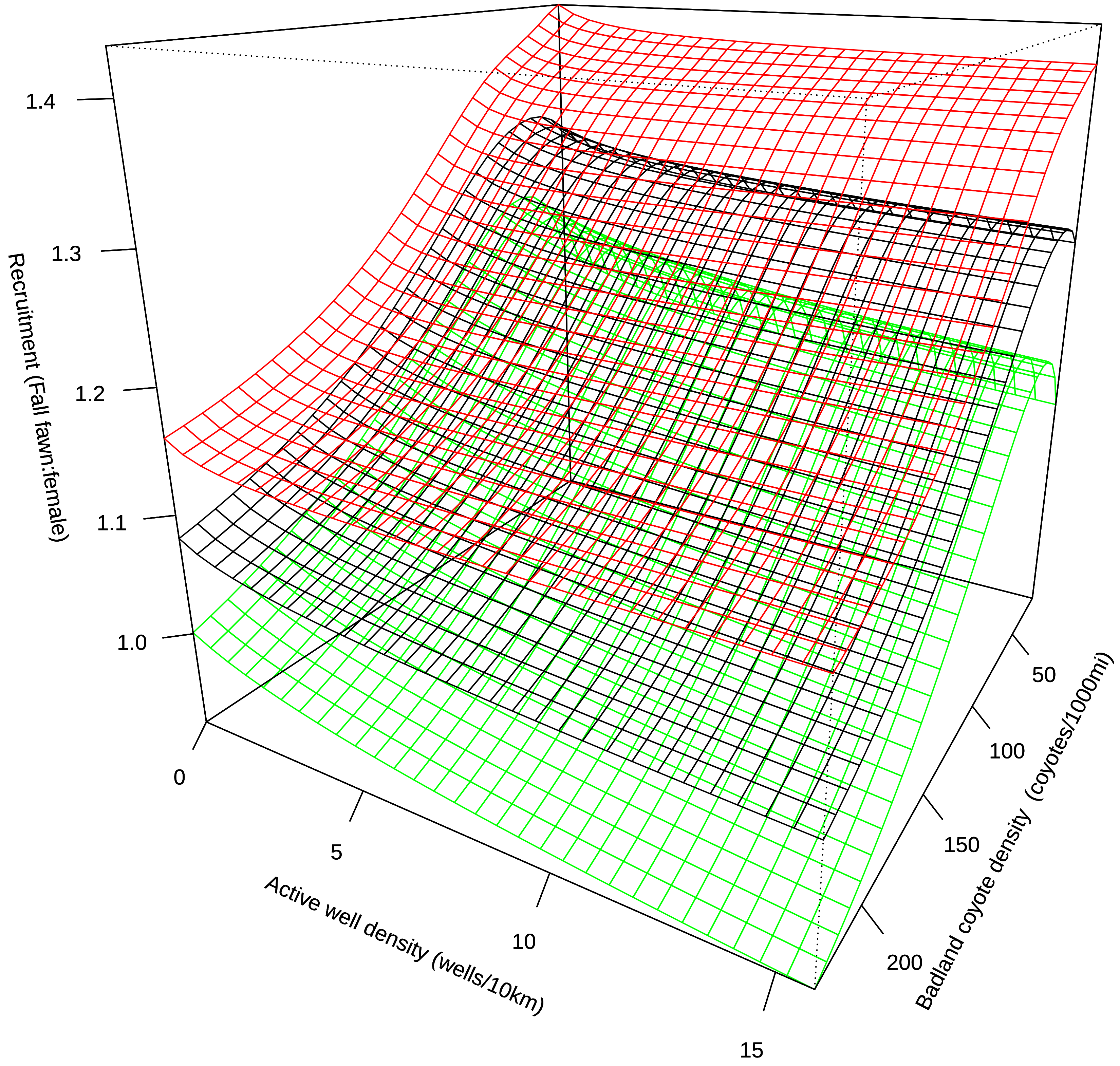

red/green are +/− 1.96 s.e.

E

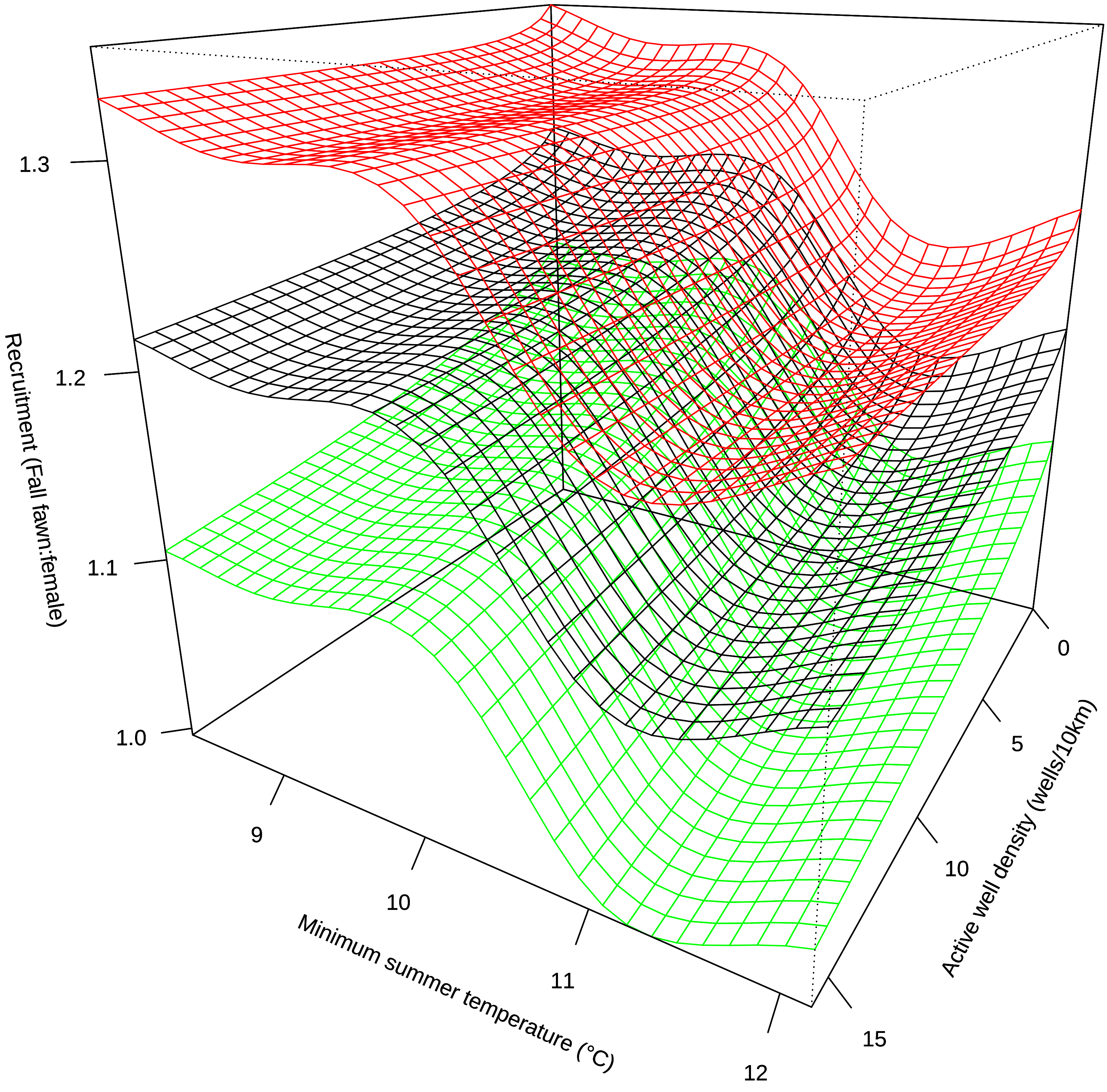

red/green are +/− 1.96 s.e.

F

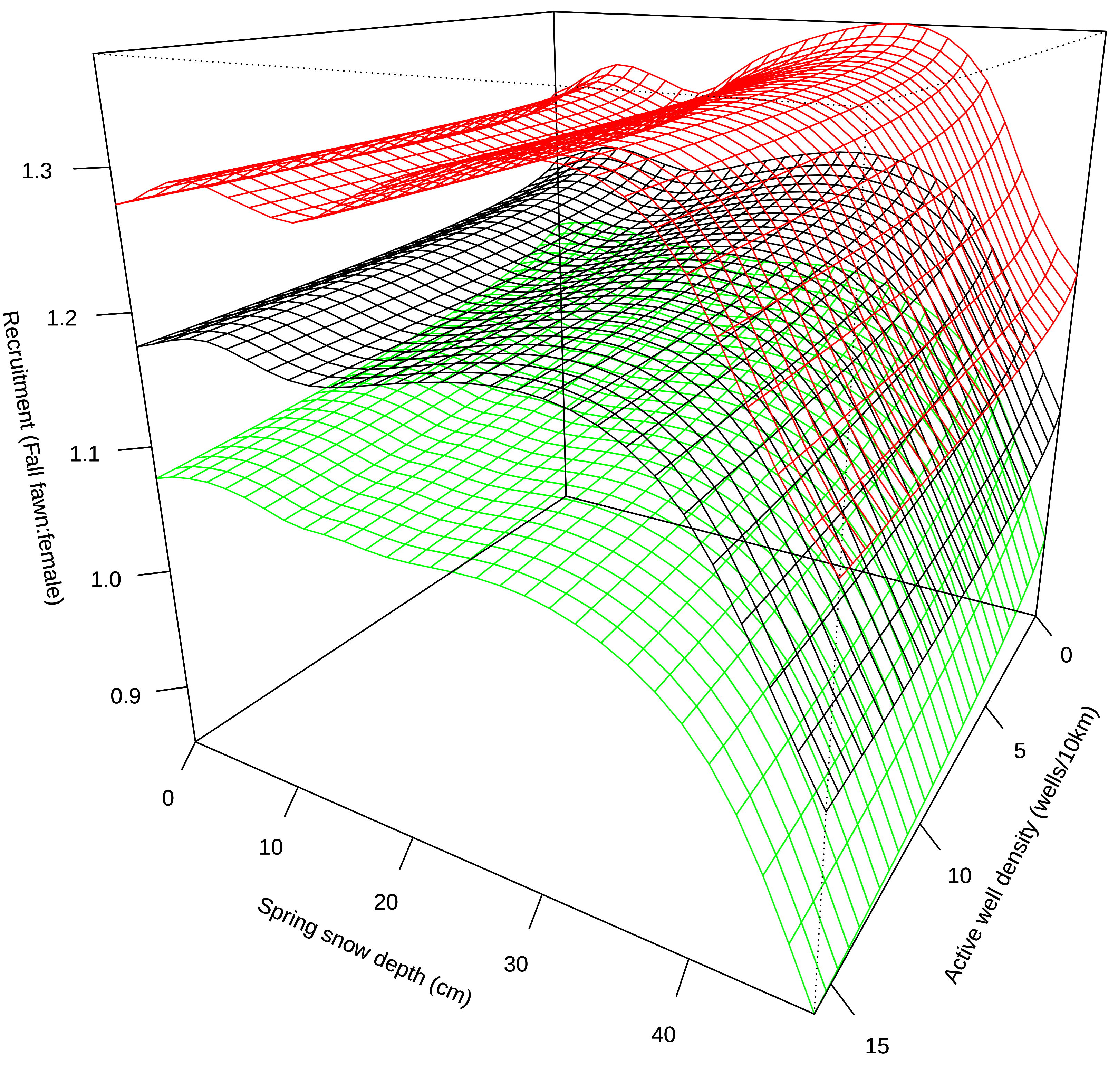

red/green are +/− 1.96 s.e.
